# Flexible Printed RFID Tag Antennas for Scalable and Sustainable IoT: Design Physics, Manufacturing Technologies, and System-Level Validation

**DOI:** 10.3390/s26196288

**Published:** 2026-10-04

**Authors:** Juan C. Rubio, Martin Bolduc

**Affiliations:** Department of Mechanical Engineering, Université du Québec à Trois-Rivières, Trois-Rivières, QC G8Z 4M3, Canada

**Keywords:** UHF RFID, printed electronics, flexible antennas, conductive inks, additive manufacturing, read range, life-cycle assessment, Internet of Things (IoT)

## Abstract

Flexible printed passive Ultra-High Frequency Radio Frequency Identification (UHF RFID) tag antennas couple electromagnetic design to conductor formation, substrate properties, chip attachment, and the mounting environment. This critical narrative review examines how those dependencies affect manufacturing choices and the strength of device-level evidence. Screen printing, inkjet printing, aerosol jet printing, flexography, and gravure are compared using reported process conditions and explicit gaps in reporting. Passive UHF tags containing an integrated circuit form the principal scope; High-Frequency/Near Field Communication (HF/NFC), chipless, and higher-frequency examples supply supporting process evidence. Given the difficulty of establishing a universal range limit across varying Equivalent Isotropically Radiated Power (EIRP) levels, chip sensitivities, polarizations, and mounting environments, this synthesis carefully categorizes performance metrics into directly measured distances, threshold-based estimates, and simulation-based predictions. Within-study comparisons show that reducing conductor consumption can sacrifice activation margin, while substrate morphology and chip bonding can limit performance even when nominal conductivity is high. Complete-tag scalability requires reproducible inter-connections, post-processing, and durability evidence in addition to successful printing. Environmental conclusions are separated into quantitative life-cycle results and indicators such as lower material use or biodegradability. The resulting framework identifies which design choices are supported by controlled comparisons, and which still require repeated device measurements, application validation, and environmental inventories matched to equivalent service.

## 1. Introduction

Ultra-high-frequency RFID has moved from being a niche identification technology to becoming one of the most operationally important infrastructures for scalable IoT in retail, logistics, and industrial environments. Its strategic value lies in the ability to assign a unique digital identity to physical objects and to connect that identity with real-time tracking, inventory visibility, event logging, and process automation across distributed supply chains [1,2,3]. Printed antennas on polymer films, paper, and textiles can accommodate thin labels and conformal integration, but their performance depends on the electrical and mechanical state of the completed tag [4,5].

Printing links antenna design to manufacturing tolerances: substrate wetting and porosity affect trace continuity, curing determines conductor consolidation, and bending or moisture can change impedance and losses (Figure 1) [4,5,6]. Consequently, a well-matched simulated layout does not by itself establish a reproducible tag. The relevant engineering question is whether a chosen material and process preserve useful RF performance after chip attachment and integration with the intended object.

The principal scope of this review is flexible printed passive UHF RFID tag antennas incorporating an integrated circuit (IC), with performance interpreted within the frequency band and application reported by each source. HF/NFC coils, chipless tags, microwave sensors, and millimeter-wave antennas are included only as supporting examples when they clarify transferable constraints related to printing, substrates, or durability. The metrics used in these supporting technologies, such as inductive coupling, spectral scattering, or conventional antenna parameters, are therefore treated separately from far-field read-range metrics for IC-based UHF RFID tags.

Prior reviews already cover printed RFID materials and techniques [4,5], general flexible antennas [7], and the integration of conductive inks, antenna architectures, manufacturing, and sustainability [8]. However, three issues remain insufficiently resolved across the literature: how process-dependent variables explain differences in reported device performance; which performance metrics and read ranges remain comparable after accounting for measurement, estimation, and test conditions; and how sustainability conclusions change when the complete tag and the corresponding assessment boundary are considered. The present review addresses these issues through a condition-aware cross-study synthesis. Table 1 summarizes how this scope extends the closest previous reviews.

Section 2 and Section 3 connect link requirements and equivalent-circuit interpretation to geometry and fabrication tolerances. Section 4 and Section 5 compare materials, substrates, and manufacturing routes. Section 6 assembles the validation evidence and a focused computational outlook; Section 7 evaluates sustainability within the boundaries of the cited studies.

### Review Scope and Evidence Selection

This article follows a critical narrative review approach. The literature was identified using Rabbit Research, SOFIA (Université du Québec à Trois-Rivières), and Google Scholar. The literature search was conducted between April and August 2026. Searches combined terms related to RFID, UHF, flexible and printed antennas, screen printing, inkjet printing, aerosol jet printing, flexography, gravure, conductivity, sheet resistance, chip attachment, read range, EIRP, bending, washing, durability, sustainability, and life-cycle assessment (LCA).

The comparative synthesis prioritized journal studies reporting relevant material or process variables together with RF, complete-tag, durability, or environmental evidence. Reviews were used for contextual support, whereas original studies were used for study-specific quantitative data. HF/NFC, chipless, and higher-frequency devices were retained only as supporting process evidence. Conference papers, patents, untraceable commercial claims, duplicated publications, and numerical results that could not be verified from the available source were excluded from the principal comparative evidence. Missing parameters are reported as “NR—Not reported.” Because device architectures and test conditions differ substantially across studies, no pooled meta-analysis was performed.

## 2. RFID Tag Antenna Fundamentals and Performance Metrics

A passive UHF tag harvests energy from the reader field to activate its IC and communicates by changing the antenna load to modulate backscatter. Successful identification requires both sufficient power at the chip and a detectable reply at the reader. Printed-conductor losses, environmental detuning, and propagation conditions can constrain these two links differently [3,9].

### 2.1. Far-Field Backscatter and Link Budget in Passive RFID

The physical principle enabling communication in passive RFID tag systems is backscatter modulation. In this scheme, the tag does not generate its own carrier as a conventional transmitter; instead, it extracts RF energy from the incident reader field to power the integrated circuit (IC) and responds by switching the load impedance presented to the antenna [10]. Figure 2 illustrates the electromagnetic operation cycle that enables this passive energy harvesting and backscatter communication process. This switching changes the reflection coefficient and therefore the backscattered power, which can be described as a controlled variation in the differential radar cross section associated with the modulated return component [11]. Information is encoded precisely in that controlled modulation of the echo.

A link budget must satisfy chip activation on the forward link and backscatter detection on the reverse link. For a far-field line-of-sight link in free space, the ideal forward-link distance is [3,12,13,14]:

(1) $$d_{tag}\;=\;\frac{\lambda }{4\pi }\sqrt{\frac{P_{EIRP}\;G_{tag}\;\tau \;\rho_{pol}}{P_{th}}}$$

Here λ is the free-space wavelength (m), $P_{EIRP}$ is the reader EIRP (W), $G_{tag}$ is the tag antenna gain in the reader direction (linear, including radiation efficiency), τ is the antenna–chip power-transfer coefficient, $P_{th}$ is the chip activation threshold (W), and $\rho_{pol}$ is the forward-link polarization-coupling factor (0–1). For matched polarization, $\rho_{\mathrm{pol}}=1$. This factor should not be applied separately when the reported gain or calibrated path loss already incorporates the same polarization loss. The expression assumes far-field line-of-sight propagation in free space, fixed reader–tag orientation, no multipath or obstruction, and a chip threshold defined for the interrogation state. It therefore represents the forward-link chip-activation limit rather than a guaranteed operational read range. If a reported realized gain already includes the same antenna–chip mismatch, τ must not be multiplied a second time.

For fixed frequency, chip threshold, polarization, and propagation conditions, Equation (1) shows that the chip-activation distance scales as $d_{\mathrm{tag}}\propto \sqrt{P_{\mathrm{EIRP}}\;G_{\mathrm{tag}}\;\tau }$. This relationship illustrates why lower conductor sheet resistance does not necessarily translate into a longer activation range: improvements in radiation efficiency may be offset by substrate-induced detuning, antenna–chip mismatch, or other losses that reduce $G_{\mathrm{tag}}\;$ or τ [8,11,12].

This dependence also highlights the importance of the experimental and regulatory conditions under which read range is reported. Radiated power must be expressed using a consistent convention when results from different studies are compared. ETSI EN 302 208 V3.4.1 specifies power levels of up to 2 W e.r.p. in the 865–868 MHz band, corresponding to approximately 33.0 dBm e.r.p. or 3.28 W (35.2 dBm) e.i.r.p. after the standard 2.15 dB conversion between the two conventions [15].

More generally, read range is a condition-specific system metric rather than a universal characteristic of the tag. Reported values vary with chip activation threshold, reader power and receiver settings, reader–tag polarization and orientation, mounting environment, propagation conditions, and measurement methodology. A threshold-based link-budget estimate may also extend beyond the largest physical separation experimentally tested. Section 6 therefore distinguishes directly measured read distances, threshold-based estimates, and simulation- or model-based predictions when comparing published performance [9,13,16].

The preceding discussion describes the forward-link chip-activation limit. A complete read-range criterion must also account for the reverse link, for which the backscattered modulation must reach the reader with sufficient power to exceed its detection threshold. For an ideal monostatic far-field return link in free space, the corresponding reader-limited distance can be expressed as:

(2) $$d_{\mathrm{reader}}={\left(\frac{P_{\mathrm{EIRP}}\;G_{\mathrm{reader}}\;\lambda^{2}\;\sigma_{d}}{{\left(4\pi \right)}^{3}\;S_{r}}\right)}^{1/4}$$

In Equation (2), $G_{\mathrm{reader}}$ is the reader antenna gain on reception (linear), $\sigma_{d}$ is the differential radar cross section associated with the detected modulation component (m^2^), and $S_{r}$ is the reader sensitivity threshold for that component (W); $P_{\mathrm{EIRP}}$ and λ retain the definitions given above. The expression assumes equal outward and return distances, far-field free-space propagation, matched polarization, and consistent modulation and radar-cross-section conventions. Receiver leakage, interference, cable losses, multipath, and fading require additional margins. Accordingly, the usable system range is constrained by $d_{system}\leq \min\left(d_{\mathrm{tag}},d_{reader}\right)$. Accordingly, demonstration of the forward-link activation threshold alone does not establish successful reverse-link detection [3,17].

Conductor loss, antenna–chip matching, and return-link detectability should therefore be evaluated separately. A favorable reflection coefficient can coexist with low radiation efficiency, while a tag may receive sufficient power to activate the chip without producing a backscattered response that exceeds the reader detection threshold [17].

### 2.2. Antenna–Chip Power Transfer and Conjugate Matching

Following the link-budget perspective introduced above, one of the main factors controlling passive UHF RFID performance is the efficiency with which the antenna transfers the harvested RF power to the integrated circuit (IC). This antenna–chip interface is governed by impedance matching. Since the chip input impedance is typically highly reactive and predominantly capacitive, the antenna must provide the complementary impedance response over the operating band. Under the classical criterion, maximum power transfer is obtained when the antenna impedance approaches the complex conjugate of the chip impedance [3,4]:

(3) $$Z_{ant}\;\approx \;{Z_{chip}}^{*}$$

Here, $Z_{ant}=R_{ant}+jX_{ant}$ is the antenna input impedance and $Z_{chip}=R_{chip}+jX_{chip}$ is the chip input impedance. The quantities $R_{ant}$ and $R_{chip}$ are their resistive components, while $X_{ant}$ and $X_{chip}$ are their reactive components, all expressed in ohms. The symbol j denotes the imaginary unit, and the superscript * denotes complex conjugation. Under ideal conjugate matching, $R_{ant}=R_{chip}$ and $X_{ant}=-X_{chip}$. The chip impedance should correspond to the received-power level and interrogation state used to define the activation threshold in Section 2.1.

The antenna–chip power-transfer coefficient τ, introduced in Equation (1), quantifies this coupling as [3,12]:

(4) $$\tau \;=\;\frac{4R_{ant}{\;R}_{chip}}{{\left|Z_{ant}\;+\;Z_{chip}\right|}^{2}}$$

For passive impedances with positive resistive components, 0≤τ≤1, with τ=1 under ideal conjugate matching. This coefficient describes power transfer at the antenna–chip interface. Radiation efficiency describes a separate process: the conversion of accepted antenna power into radiated power.

Matching can be implemented through printed features such as a T-match, an inductively coupled loop, a meandered trace, a slot, or a capacitive gap [3,12]. Near a dominant series-type resonance, a representative equivalent circuit referred to the antenna terminals expresses the input impedance as:

(5) $$Z_{ant}(\omega )\;\approx \;R_{rad}\;+\;R_{loss}\;+\;j(\omega L_{eff}\;-\;\frac{1}{\omega C_{eff}})$$

Here, ω=2πf is the angular frequency (rad/s), with f denoting frequency (Hz). The equivalent radiation resistance $R_{rad}$ represents radiated power, and $R_{loss}$ represents conductor and dielectric dissipation, both in ohms. The effective inductance $L_{eff}$(H) and capacitance $C_{eff}$(F) represent the distributed magnetic- and electric-energy storage associated with the layout. Within this representation, $R_{ant}=R_{rad}+R_{loss}$. The circuit provides a local approximation over a specified frequency interval; coupled-loop and T-match structures can require mutual-inductance or transformer terms, while other resonant responses can require parallel branches [3,12,18]. Figure 3 summarizes this local equivalent-circuit interpretation and illustrates how the principal printed-layout and material parameters are associated with the resistive and reactive terms governing antenna–chip matching and radiation.

The connection to printing tolerances can first be illustrated through conductor resistance. For a uniform trace in the direct-current (DC) limit, $R_{trace,DC}\approx R_{s}l/w$, where $R_{trace,DC}$ is the trace resistance (Ω), $R_{s}$ is the DC sheet resistance (Ω/□), and l and w are the trace length and width (m). Under a homogeneous-film approximation, $R_{s}\approx 1/\left(\sigma h\right)$, where σ is the effective electrical conductivity of the processed film (S/m) and h is its thickness (m). Narrower or longer traces therefore increase resistance, while reduced conductivity or thickness increases sheet resistance. At RF, the conductor contribution to $R_{loss}$ additionally depends on skin effect, current crowding, and the spatial distribution of antenna current [8].

Geometric changes also modify the reactive terms. For otherwise comparable geometry and dielectric surroundings, narrowing a capacitive gap increases local capacitance, while lengthening an inductive current path generally increases its inductive contribution. In closely spaced meanders or coupled loops, mutual coupling further modifies the effective response. Changes in trace width, gap size, and current-path length also redistribute current and charge, affecting $R_{rad}$ and the radiation pattern. Slot and capacitively loaded structures provide examples of this connection between local geometry, stored energy, and impedance [3,18].

Within the same input-referred representation, the radiation efficiency is $\eta_{rad}=R_{rad}/\left(R_{rad}+R_{loss}\right)$, where $\eta_{rad}$ denotes the fraction of accepted antenna power that is radiated. A fabrication-induced change can bring the antenna impedance closer to the chip conjugate while increasing dissipative resistance, allowing τ to improve as $\eta_{rad}$ decreases. The circuit parameters should therefore be evaluated over the intended operating band. Impedance measurements or full-wave simulations can constrain the combined input resistance and reactance; separating $R_{rad}$ from $R_{loss}$ additionally requires radiation-efficiency information or calculations of radiated and dissipated power. This distinction connects printing-induced geometric and material variations to their respective effects on power transfer and radiation [3,12,18].

### 2.3. Antenna Parameters That Dominate Read Range

For fixed chip sensitivity and EIRP, the useful design target is the combination of directional gain, matching, bandwidth, and stability under the intended mounting and deformation conditions. Thickness and conductor consolidation affect RF loss; substrate permittivity and geometry affect both matching and the radiation pattern [4,8].

Radiation efficiency, the fraction of accepted antenna power that is radiated, is sensitive to the electrical quality of the printed conductor. Sheet resistance, finite thickness and surface roughness affect dissipative losses. At UHF frequencies, conductor thickness must be interpreted relative to the skin depth *δ* [8]:

(6) $$\;\delta =\sqrt{\frac{1}{\pi f\mu \sigma }}$$

Here, δ is the skin depth (m), and μ is the magnetic permeability of the conductor (H/m). Equation (6) assumes sinusoidal steady-state excitation and a homogeneous, isotropic conductor in which conduction current dominates displacement current, with real-valued permeability and conductivity evaluated at the operating frequency. A DC-derived conductivity provides an approximation when the processed film exhibits sufficiently weak frequency dependence over the band [4,8].

Comparing the deposited thickness h with δ helps identify the regime in which current penetrates a substantial fraction of the film. The heuristic h≳3δ provides a reference for approaching thick-conductor behavior in smooth, homogeneous materials. For particulate printed films, its interpretation also requires consideration of porosity, particle connectivity, anisotropy, surface roughness, and local discontinuities. These features motivate the combined use of thickness measurements, DC sheet resistance, and RF performance characterization when assessing conductor quality [4,8].

Miniaturization introduces a related trade-off between stored energy and usable bandwidth. In low-loss, single-resonance designs, reducing electrical size commonly increases the quality factor Q, a dimensionless measure relating stored electromagnetic energy to energy radiated and dissipated per cycle. A higher Q generally produces a narrower impedance response for a given matching criterion, increasing sensitivity to geometric tolerances and environmental detuning. Maksimenko et al. illustrated these bandwidth constraints in compact ceramic UHF RFID tags [18]. For flexible printed tags, usable bandwidth should consequently be evaluated through the antenna–chip power-transfer or read-range requirement across the intended frequency band.

Orientation and mounting conditions determine how these antenna properties translate into practical coverage. Dobrykh et al. demonstrated a mechanically self-aligning tag that maintains a favorable orientation relative to the reader field, illustrating an approach to mitigating polarization mismatch [13]. Murugesh et al. reported an on-metal folded-patch design with embedded capacitance and a ground structure, combining frequency tuning with reduced sensitivity to the backing object and polarization variations [19]. These architectures provide supporting evidence for the roles of current distribution, electromagnetic coupling, and mounting conditions in tag performance.

For flexible printed implementations, performance assessment should therefore cover the specified frequency band, relevant reader–tag orientations, representative mounting objects, and intended bending or environmental exposure. Reporting the minimum read range achieved over these defined conditions, together with the corresponding test settings and sample variability, makes the available performance margin explicit. This provides a practical basis for relating geometric and material tolerances to reproducible tag operation [9,10,20].

## 3. Design Physics of RFID Tag Antennas

### 3.1. Geometry-Driven Matching and Miniaturization

Geometry redistributes current paths and stores electric and magnetic energy within the available footprint. The equivalent-circuit interpretation in Section 2.2 links these geometric features to impedance matching, conductor losses, and radiation efficiency, while the selected printing process constrains the minimum reproducible gaps, trace widths, and film thicknesses [3,18].

Folded and meandered dipoles reduce the external dimensions by extending the current path inside a smaller area, often with an inductive loop or T-match at the feed (Figure 4). The benefit is a planar layout; the cost can be current cancellation, additional conductor loss, narrow bandwidth, and greater sensitivity to inter-trace capacitance. Accordingly, miniaturization must preserve the conjugate-matching and power-transfer conditions represented by Equations (3) and (4), together with adequate radiation efficiency [8,18,21].

El Ahmar et al. [21] provide a flexible inkjet-printed example combining meander-line compression and split-ring loading on polyimide. Their reader test demonstrated operation at 4 m with a foam-mounted tag and a linearly polarized reader antenna. The source separately predicts approximately 4.5 m using a stated 500 mW EIRP, while describing reader maximum power and gain without establishing an equivalent calibrated EIRP for the distance test. These results establish a fabricated functional tag, but the predicted and tested ranges should not be treated as interchangeable or used to rank printing routes.

Miniaturization also increases the importance of stored reactive energy. For an electrically small antenna represented by a dominant dipolar spherical mode, the Chu–Harrington expression supplies a useful, idealized quality-factor bound [18]:

(7) $$\;Q\;\geq \;\frac{1}{kR}\;+\;\frac{1}{{\left(kR\right)}^{3}}$$

Here *k* = 2π/λ and *R* is the radius of the smallest enclosing sphere. This idealized bound is a design warning, not an exact bandwidth prediction for every lossy, dielectric-loaded, multiresonant printed tag. Loss can broaden an impedance response while reducing efficiency, so a wider measured bandwidth is not necessarily evidence of better radiation. The ceramic study [18] supplies a physical miniaturization reference; fabrication tolerance and mounting tests remain necessary for flexible printed implementations.

The appropriate topology therefore depends on the limiting constraint. A simple folded or meandered dipole may suit a thin label when moderate gaps and continuous traces can be printed reproducibly. A grounded patch or isolated structure can better tolerate metal or the human body, at the expense of thickness, assembly steps, and potentially reduced conformity [11,22,23].

### 3.2. Environment-Aware and Platform-Tolerant Design

Mounting changes the electromagnetic boundary condition. Metal, water-rich products, and the human body can change impedance, dissipative loss, and radiation-pattern shape, affecting both activation and backscatter. Platform tolerance must therefore be evaluated on defined objects, with their dimensions and spacing reported [11,23,24].

Ground planes, shorted structures, and electromagnetic isolation layers reduce coupling to the supporting object. Electromagnetically, these structures modify the field distribution and current return path, reducing the sensitivity of the radiator to the electrical properties of the underlying platform. These approaches can preserve matching, but the complete stack must still meet the thickness and conformity requirements of the application; a flexible radiator on a thick spacer is not equivalent to a thin conformal label [11,25].

Slot tuning and isolation address different failure mechanisms. Slots primarily reshape the impedance response, whereas a ground plane or artificial magnetic conductor (AMC) changes coupling to the mounting surface. Combining them may improve robustness, but it also introduces additional dimensions, and material interfaces whose tolerances must be controlled [22,24].

Bansal et al. [23] measured read distance by moving the tag away from a circularly polarized reader in an outdoor setting: 5.9 m on metal, 7.1 m on an ABS helmet, 7.0 m on plastic, 4.9 m on foam, 6.1 m on wood, and 9.3 m on glass. These measurements provide useful evidence of platform-dependent performance under practical mounting conditions. However, the assembled three-dimensional patch architecture is better interpreted as a platform-tolerant implementation than as a printed-film manufacturing benchmark; the mounting comparison also combines changes in impedance, gain, and radiation efficiency rather than isolating the effect of substrate material alone.

Casula et al. [22] report calculated read ranges of approximately 5.5 m on low-permittivity dielectrics, 8 m on a body model, and 11 m on metal, using simulated gain and matching with a specified reader and chip model. These values are theoretical system predictions rather than direct distance measurements on the corresponding objects. Their AMC-backed design illustrates how electromagnetic isolation can improve radiation performance under strongly loading platforms, but the predicted ranges should be interpreted separately from the outdoor distance measurements reported by Bansal et al. [23].

The comparison therefore separates two questions: whether an isolation mechanism provides the intended electromagnetic benefit, and whether the manufactured tag preserves that benefit after fabrication and deployment. The latter additionally requires evidence from repeated assemblies, interlayer registration, bending, and measurements on representative mounting objects. Section 6 uses these distinctions to classify the available studies.

### 3.3. Design Implications for Scalable Printed RFID

A manufacturable printed design begins with the operating band, chip impedance and activation threshold, mounting stack, and required reading function. Process selection then constrains the achievable trace width, gap size, deposited thickness, curing temperature, conductivity, and registration accuracy [4,8].

Optimization should evaluate a tolerance interval rather than only nominal dimensions. A slightly larger layout can be preferable if it retains activation margin under realistic changes in line width, conductor consolidation, or substrate moisture, whereas a compact design based on exceptionally narrow gaps or tightly controlled reactive features may be difficult to reproduce consistently [8,18].

This creates a process-dependent manufacturability window in which electromagnetic performance and fabrication capability must be considered together. From a review perspective, the relevant distinction is therefore whether a study reports only nominal simulated performance or also demonstrates robustness to dimensional variation, material variability, and fabrication repeatability. Designs that retain acceptable matching and radiation efficiency across these variations provide stronger evidence of scalability than designs optimized around a single nominal geometry.

The final design decision should be based on completed tags. Conductor and interconnect resistance should be characterized, RF matching established, activation threshold measured, and readability evaluated under the intended mounting and mechanical conditions. The manufacturing and validation comparisons in Section 5 and Section 6 identify where these steps are supported experimentally and where the available reporting remains incomplete.

## 4. Conductive Inks and Substrates for Printed RFID Tag Antennas

Material selection in printed RFID antennas is governed by the electrical, dielectric, and interfacial behavior of the complete conductor–substrate system. Conductivity, sheet resistance, deposited thickness, curing conditions, substrate permittivity, surface characteristics, and adhesion collectively influence RF losses, dimensional stability, and manufacturing compatibility [2,26]. This section examines conductive inks and flexible substrates as a coupled materials stack, with emphasis on how their reported properties are obtained, how they translate to completed antennas, and which material–process trade-offs are most relevant for scalable printed RFID.

### 4.1. Conductive Inks for Printed RFID Antennas

The electrical performance of conductive inks is most meaningfully interpreted from the properties of the consolidated film under its specific deposition and post-processing conditions [4,26]. Table 2 therefore compares representative conductor systems together with the substrate, dry-film thickness, sheet resistance, conductivity, and provenance of the reported electrical values.

For Ag nanoparticle inks, reported film properties must be separated from properties measured on the final antenna. In the journal study by Chietera et al. [27], the 24.2 mΩ/sq value corresponds to an 18 µm reference film on glass; it is not a measured sheet resistance of the antenna deposited on the printed ABS support. Similarly, the cited 10^5^–10^7^ S/m conductivity interval is process-dependent and should not be assigned to every printed tag. The approximately 7 m AJP and 3 m filament-printed results are maximum-range estimates obtained from RF threshold measurements. Their comparison supports a conductor-processing trade-off within that experimental design, rather than a universal ranking of the manufacturing methods.

The apparent inconsistency in the silver-ink comparison arises from different substrates and film conditions. Machiels et al. [26] report less than 5 mΩ/sq for both Orgacon SI-P2000 and Loctite ECI 1011 on PET after 150 °C curing for 10 min. On the selected paper substrates, Loctite instead produced 10–34 mΩ/sq; on Magno Satin, the measured values were 28 ± 6 mΩ/sq for Orgacon and 32 ± 2 mΩ/sq for Loctite. Nevertheless, complete HF antennas made with Loctite functioned, whereas the tested Orgacon implementation did not: their series resistances were 22.1 ± 2.3 Ω and 38.2 ± 1.9 Ω, respectively. Coupon sheet resistance therefore cannot replace validation of the printed geometry, inductance, and chip interface. The conductivity values previously inferred from nominal resistance at a reference thickness are not presented as measurements of these paper-supported films.

Ti_3_C_2_T_x_ MXene is a transition-metal carbide, distinct from graphene and other carbon conductors. Sourkouhi et al. [28] reported approximately 8.6 µm thick MXene coatings and local sheet resistances of 0.3–1.6 Ω/sq. A conductivity of 1.3 × 10^6^ S/m was used in the antenna simulations and should not be conflated with a spatially uniform measured film property. The approximately 97% result describes an impedance-based power-transfer coefficient, not radiation efficiency or a percentage of copper-antenna efficiency. The dipole and meander structures were matched to 50 Ω, whereas the T-matched design targeted a complex chip impedance. Integration with a repurposed commercial IC demonstrated communication at approximately 0.5 m; the authors identified residual chip–antenna mismatch. Room-temperature processing is a favorable process attribute, but this study does not establish a lower life-cycle environmental impact.

Bioresorbable systems also require a distinction between antenna conductors and supporting functional coatings. In Say et al. [29], HEC/Mo and HEC/W composites provide electromagnetic shielding around an ingestible capsule; the RFID antenna itself uses zinc foil on cellulose acetate. The reported 0.8 Ω/sq HEC/Mo value therefore characterizes a shielding coating and cannot be used to rank printed antenna conductors. Dissolution in physiological media demonstrates a specific end-of-life mechanism, while the silicon chip remains a separate component; neither observation constitutes a comparative life-cycle assessment.

Material selection should consequently use matched combinations of sheet resistance, dry thickness, substrate, and post-processing, with the provenance of conductivity values identified explicitly. Silver-film results demonstrate mature low-resistance processing, whereas graphene and MXene examples reveal different thermal, integration, and stability constraints. A lower nominal conductivity may be acceptable when layout and available link margin tolerate the added loss, but material composition alone establishes neither tag performance nor environmental superiority.

### 4.2. Substrates for RFID Tag Antennas

Substrate selection affects both dielectric loading and conductor formation. Table 3 records source-specific thickness, dielectric parameters, surface or moisture behavior, processing conditions, and mechanical evidence. The listed curing conditions are demonstrated process settings, not certified maximum-use temperatures; missing values are not replaced by generic handbook properties.

The flexibility of a carrier film does not establish the flexibility of the complete mounted tag. A thick dielectric spacer can reduce metal-induced detuning while increasing assembly thickness and limiting conformity. Substrate comparison should therefore distinguish the primary carrier, protective or interface layers, and mounting spacer, and should report total thickness and mechanical test conditions for the complete assembly. Table 3 separates source-reported material properties from demonstrated device behavior and identifies missing information as NR.

Kaur et al. [30] are particularly relevant to substrate selection because their results separate the role of the flexible carrier from that of the complete metal-mount stack. The 125 µm PET film provides a thin and flexible support for the printed Ag antenna, whereas tolerance to the metallic platform is achieved primarily through dielectric separation. The comparison between silicone (εr = 4) and Styrofoam (ε_r_ = 1) shows that spacer permittivity and thickness jointly control the recovery of tag performance near metal: approximately 20 mm of silicone produced performance comparable to about 40 mm of Styrofoam under the reported test conditions. This result highlights a practical substrate-design trade-off: stronger electromagnetic isolation can be obtained through the spacer, but at the cost of increased assembly thickness.

The same study also illustrates why “flexible substrate” and “conformal tag” should be treated as different engineering attributes. Although the PET carrier is flexible and the silicone layer is elastomeric, the final industrial prototype employed a 40 mm spacer and additional ABS or polyamide encapsulation, yielding a metal-mount structure with a reported depth of 40 mm. Its read performance on metallic stillages therefore provides strong evidence of platform tolerance, while offering limited evidence of thin-label conformity or repeated-flexibility performance. The authors’ own identification of spacer-thickness reduction as future work reinforces thickness as a remaining integration constraint rather than a secondary packaging detail.

**Table 3:** Study-specific substrate properties and evidence.

Substrate/Study	Thickness	Dielectric Properties/Frequency Context	Surface/Moisture Evidence	Thermal/Mechanical Evidence
Magno Satin coated paper [26]	Not reported (NR)	ε_r_ and tanδ: NR; HF study	Ra = 424 ± 24 nm; air permeance 0.2 ± 0.1 mL/min; moisture-dependent RF data NR	150 °C/10 min curing; moisture-related curl observed
Patterned glossy paper [28]	Carrier thickness NR; superhydrophobic coating ≈ 45 µm average; MXene coating ≈ 8.6 µm	ε_r_ and tanδ: NR; 800–920 MHz designs	Static contact angle > 150° on the superhydrophobic region and <90° on hydrophilic traces; source-reported advancing/receding angles are 153.4°/157.2° for the superhydrophobic region and 45.4°/25.4° for the MXene traces; superhydrophobic coating Rp ≈ 59 µm and dried MXene trace Rp ≈ 55.14 µm; moisture-response data NR	≈22 °C/24 h drying; three antennas manually rolled/deformed; <1.5% variation in maximum power-transfer coefficient after bending; bend radius and cycle count NR
PET [31]	135 µm	ε_r_ = 3, tanδ = 0.002 as design inputs; 922.5 MHz comparison	Not reported	Curing and cyclic mechanical tests NR
Polyimide [32]	Carrier NR; copper trace 35 µm	ε_r_ = 3.4, tanδ = 0.0025 used for the 868–928 MHz design	Not reported	PLA-channel integration; cyclic mechanical test NR
Woven polyester [33]	Plain/twill/satin: 0.252/0.322/0.372 mm	Study-specific dielectric comparison; no generic value assigned	Weave controls porosity, roughness and conductor spreading	UV curing for 40 s; five repeat electrical tests; bending/washing NR
Polyester-coated nylon [34]	0.114 ± 0.001 mm	ε_r_ and tanδ: NR	Compressibility and coating affect chip contact	120 °C/30 min conductor cure; bonding parameters investigated
Cellulose acetate [29]	35 µm	ε_r_ and tanδ: NR; 915 MHz foil antenna	Gastric-media dissolution; ambient humidity behavior NR	37 °C simulated/real gastric fluids; general cure limit NR
PET carrier + silicone spacer [30]	PET carrier: 125 µm; Ag conductor: 20 µm; proposed silicone spacer: 10–40 mm; spacer experiments: 10–50 mm; final metal-mount prototypes: 40 mm silicone	Silicone ε_r_ = 4; Styrofoam ε_r_ = 1 used as a spacer comparator; UHF tests included operation around 866 MHz	Surface roughness and moisture response NR	PET selected as a flexible carrier; silicone used as an elastomeric spacer. No bend radius or cyclic-flexibility test of the complete mounted assembly was reported. Final 40 mm prototypes were additionally encapsulated in ABS or polyamide

Cellulose acetate represents a different substrate-selection problem. In Say et al. [29], the value of the 35 µm support lies in its compatibility with a transient device intended for gastric dissolution rather than in conventional long-term environmental stability. The study therefore provides application-specific evidence for bioresorbable RFID, but it should not be generalized to outdoor biodegradation, recyclability of the assembled tag, or reduced life-cycle impact. In this context, substrate suitability is defined by controlled disappearance in the intended environment rather than by durability or reuse.

Sourkouhi et al. [28] introduce another critical dimension, showing that substrate surface chemistry can become an active manufacturing variable rather than simply a dielectric boundary. Patterning relies on a strong wettability contrast between the superhydrophobic and hydrophilic regions, which confines the aqueous MXene dispersion without a conventional removable printing mask during deposition. The source reports static contact angles above 150° for the superhydrophobic region and below 90° for the hydrophilic region, followed by advancing/receding values of 153.4°/157.2° and 45.4°/25.4° for the superhydrophobic and MXene-coated regions, respectively. Because the reported receding angle exceeds the advancing angle for the superhydrophobic region, those values are best retained as source-reported rather than reinterpreted or interchanged. The engineering implication is nevertheless clear: wettability control directly affects liquid confinement and therefore pattern fidelity.

The same work also demonstrates why surface engineering should be assessed together with conductor uniformity. The superhydrophobic coating had an average thickness of approximately 45 µm, while the dried MXene layer was approximately 8.6 µm thick; local roughness and paper porosity contributed to variations in the deposited trace. Sheet resistance varied from approximately 0.3 to 1.6 Ω/sq across fabricated traces, and the authors attributed part of that spread to substrate characteristics and local MXene flake alignment. This limits the usefulness of treating “paper” as a single homogeneous substrate category: its porosity, coating state, and local topography can directly affect electrical reproducibility.

Mechanical evidence from Sourkouhi et al. [28] is informative but should also be interpreted within the test protocol. Three paper-based dipole antennas were manually rolled and deformed, after which impedance and power-transfer measurements were repeated. The maximum power-transfer coefficient changed by less than 1.5%, and the sheet-resistance measurements remained consistent after deformation. This supports short-term deformation tolerance of the demonstrated structures. However, no standardized bending radius or cycle count was reported, so the study does not establish cyclic mechanical durability or fatigue resistance.

Textile and leather-like substrates introduce a different design constraint because the host material itself becomes part of the mechanical and electromagnetic environment. Abdulghafor et al. [35] demonstrated a multitexture UHF RFID sensing platform for wearable hydration monitoring, whereas Hanif et al. [36] used a flexible Rexin artificial-leather substrate for a wearable UHF RFID logo tag. Together, these studies show that wearable integration can be achieved on deformable surfaces, but they also highlight the need to report coating penetration, moisture sensitivity, chip-junction stability, and deformation conditions. Results obtained on a specific textile architecture or artificial-leather formulation should therefore be interpreted as application-specific evidence rather than as general properties transferable to all wearable substrates.

Polyimide provides a useful dielectric reference when its properties are tied to the implementation. Ennasar et al. [32] used ε_r_ = 3.4 and tanδ = 0.0025 for a copper-trace UHF sensing antenna designed over 868–928 MHz. These values are meaningful within that design context, but they should not be treated as universal, frequency-independent properties of all polyimide grades. The integrated PLA fluid channel also forms part of the mechanical stack, so carrier flexibility and complete-device conformity should be evaluated separately.

Taken together, the studies in Table 3 show that substrate selection is best treated as a conditional engineering decision rather than as a ranking of material families. Smooth polymer films can simplify dimensional and thickness control, whereas paper and textiles may require surface treatment, coating control, or interface layers that modify both electrical and mechanical behavior. Metal-mount applications may require thick dielectric spacing that restores RF performance while penalizing total thickness and conformity. Bioresorbable systems introduce yet another criterion, in which controlled degradation becomes a functional requirement. Across these cases, the most informative studies are those that report dielectric properties together with thickness, surface condition, processing limits, and mechanical test conditions for the actual assembled tag rather than for the carrier material alone.

## 5. Manufacturing Technologies

The manufacturing routes reviewed below are evaluated using both process-specific evidence and a common cross-process framework. Table 4 and Table 5 retain source-level details for representative screen- and inkjet-printed structures, while Table 6 consolidates screen printing, inkjet printing, aerosol jet printing, flexography, and gravure using demonstrated feature size, dry-film thickness, sheet resistance, substrate and post-processing constraints, reported production rate, reproducibility evidence, and conditional selection rules.

### 5.1. Screen Printing

Screen printing transfers a viscous functional ink through a patterned mesh. Its practical advantages are the ability to deposit comparatively thick layers and to accommodate paper, polymers, and textiles. Mesh geometry, ink rheology, off-contact distance, squeegee conditions, and curing jointly determine trace width and film continuity [37,38]. Table 4 reports source-specific process settings and separates UHF tag studies from supporting RF examples.

**Table 4:** Screen-printing conditions and device evidence.

Device/Study	Conductor; Substrate	Printing Conditions	Dry Layer/Resistance	Post-Processing	Evidence Limitation
UHF textile [33]	UV-curable Ag ink, 60 wt%; polyester plain/twill/satin	170 mm/s; 85° squeegee	0.2180/0.1508/0.1445 mm, respectively; measured	UV curing, 40 s	Five repeat electrical tests; substrate-specific conductor thickness and resistance
Passive UHF fabric [34]	ET-4F Ag, 65%; coated nylon, 0.114 ± 0.001 mm	300 mesh; 4 mm off-contact; 85°; 170 mm/s	3.4 ± 0.2 µm; Rs NR	120 °C, 30 min; ACA flip-chip attachment	Bonding alters the conductor and chip contact
5.8 GHz wearable antenna comparator [39]	HPS-FG32 Ag ink; 82% nylon/18% spandex	230 mesh	Conductor thickness NR; interface layer ≈ 100 µm	Interface layer added to reduce ink penetration; washing up to five cycles	Supporting textile fabrication/durability evidence; not UHF RFID range evidence
915 MHz wearable UHF RFID [39]	NovaCentrix FLX15 Ag ink; 60% cotton/40% viscose	156 mesh; NXP UCODE G2iL attached with conductive adhesive	Conductor thickness NR; sheet resistance in standard Ω/sq units NR	Mounted on skin-mimicking gel for testing	Three tags tested; direct corridor read distances 1.4–2.6 m (mean 2.2 m); independent-batch production yield NR
Near-field UHF [40]	Graphene; PI for final tag	200 tpi; three passes; ≈100 µm line width	135 Ω/sq before; <10 Ω/sq after post-processing	100 °C/10 min per pass; up to 320 °C/36 min; compression: 4 passes, 80 bar, 2 m/min	Fine chip-contact gaps are sensitive to repeated-print registration
UHF transponder [16]	Few-layer graphene; Kapton	90 mesh/in; viscosity 570 mPa·s at 100 s^−1^ and 140 mPa·s at 1000 s^−1^	Thickness NR; Rs ≈ 5 to ≈3 Ω/sq	265 °C, 10 min	More severe annealing caused delamination
915 MHz sensor [41]	AG-510 silver; paper	Laser-cut wax-paper stencil; blade deposition	Thickness and Rs NR	60 °C until dry	Stencil process; production throughput NR
Chipless comparator [42]	ESL 1901-S Ag; 125 µm polyester or 100 µm paper	Semi-automatic Aurel C880; three print repetitions	30 µm; Rs < 0.020 Ω/sq reported on test track	80 °C, 30 min	RCS validation; not an IC-based UHF tag
HF [26]	Orgacon/Loctite; Magno Satin paper	Antenna screen: 140 threads/cm; 5 mm off-contact; 100 mm/s; 16°; 66 N	Paper coupons: 6–7 µm; Rs 0.028 ± 0.006/0.032 ± 0.002 Ω/sq	150 °C, 10 min	Coupon properties and full-antenna functionality differ

The textile studies collectively show that screen-printing performance is governed by a substrate-dependent process window rather than by nominal printing settings alone. Zheng et al. [33] used the same printing speed and scraper angle across woven polyester structures, but linked changes in antenna behavior to roughness, porosity, and wetting. Compared with smooth-film printing, the additional uncontrolled variable is the fraction of ink retained as a continuous surface trace rather than absorbed into the fabric (Figure 5a). This makes substrate morphology a manufacturing variable in its own right: identical printer settings can produce electrically different conductors when the ink–substrate interaction changes.

Complete-tag manufacture includes chip attachment as well as conductor deposition. Meng and Hu [34] printed a 3.4 ± 0.2 µm silver layer on coated nylon and cured it at 120 °C for 30 min. Subsequent anisotropic-conductive-adhesive bonding altered the local conductor structure and the electrical contact: insufficient pressure impaired contact, whereas excessive pressure could damage the printed track. This study therefore exposes a different limitation from that identified by Zheng [33]: once conductor formation is adequately controlled, the dominant manufacturing constraint can move to the chip–antenna interconnection, where bonding pressure, contact resistance, and local conductor integrity become critical.

The reported thickness cannot be assessed against a bulk-silver skin depth without establishing the effective conductivity of the printed film. Manufacturing validation should therefore distinguish conductor continuity, chip-contact resistance, successful activation, and interface integrity after deformation. Accordingly, yield should be defined at completed-tag level rather than from the fraction of visually intact printed antennas. Encapsulation or local protection of the chip–antenna junction should likewise be included in complete-tag manufacturing assessment, since the final protective stack can influence moisture exposure, adhesion, strain transfer, and RF detuning. Scalability should therefore be evaluated after chip attachment and final protection, using the assembled tag as the manufacturing unit.

Hasni et al. [39] provide a complementary example in which the substrate problem was mitigated by modifying the stack itself. An approximately 100 µm non-conductive interface layer reduced silver-ink penetration into the fabric, but the added dielectric also shifted the resonance and reduced flexibility as its thickness increased. The result illustrates a recurring textile trade-off: an interface layer can improve conductor localization while simultaneously introducing electromagnetic and mechanical penalties. The 5.8 GHz textile antennas retained resonance after five washing cycles, which provides useful durability evidence for the fabrication concept but should remain separate from UHF RFID range performance. In the same study, a separate 915 MHz UHF RFID implementation was screen printed on 60% cotton/40% viscose using a 156-mesh screen and NovaCentrix FLX15 ink. Three tags mounted on skin-mimicking gel achieved direct corridor read distances of 1.4–2.6 m, with an average of 2.2 m. Because these distances were obtained under body-like loading, they are more useful as evidence of application-specific operability than as a general performance limit for screen-printed UHF tags.

Carbon-based conductors reveal a different set of trade-offs. In Jaakkola et al. [40] screen printing of graphene on flexible substrates was feasible because the technique can handle viscous, high-solid-content inks and generate thick films, but acceptable RFID performance required aggressive post-processing. Their near-field graphene antennas were printed with a 200 threads-per-inch screen in three passes, each followed by 100 °C/10 min drying; then, for PI-based samples, thermal annealing up to 320 °C for 36 min and rolling compression (4 passes, 80 bar, 2 m/min) reduced sheet resistance from 135 Ω/sq to <10 Ω/sq. At the same time, the authors noted that multilayer printing increases the risk of mismatch and shorts near the chip-contact region, especially when dimensions fall below 200 μm. A similar compromise appears in the graphene transponder study in 2D Materials, where screen printing on Kapton used a 90 mesh screen and subsequent 265 °C/10 min annealing to reduce sheet resistance from about 5 Ω/sq to 3 Ω/sq, but stronger annealing led to delamination from Kapton [16].

Taken together, these graphene studies show that the ability of screen printing to deposit comparatively thick films does not remove the conductivity problem; instead, it can transfer the manufacturing burden to thermal consolidation and adhesion. The relevant selection criterion is therefore whether the substrate can tolerate the post-processing required to reach the target electrical resistance without loss of dimensional or interfacial integrity.

The silver-on-paper implementation reported by Wang et al. [41] represents a comparatively low-thermal-budget screen/stencil processing route. The 915 MHz flexible RFID sensor was fabricated using AG-510 silver ink followed by thermal drying on a hot plate at 60 °C, demonstrating that functional paper-based RFID structures can be realized without the high-temperature annealing required by several graphene systems. The principal trade-off is a narrower manufacturing margin with respect to dimensional fidelity, conductor continuity, passive-component tolerances, and print-induced electrical variability. From a process-selection perspective, such low-temperature routes are particularly attractive when substrate thermal compatibility and manufacturing simplicity are dominant constraints. By contrast, applications requiring lower conductor resistance and tighter RF tolerances may benefit from more intensive thermal or mechanical consolidation, if adhesion, substrate dimensional stability, and interfacial integrity are preserved.

Divya and Lee [43] used screen printing to define an aluminum precursor on PET and then formed a copper conductor by galvanic displacement (Figure 5d). This hybrid route demonstrates that the printed material need not be the final radiator. More importantly for process selection, it decouples geometric pattern formation from final conductor formation: screen printing defines the layout, whereas the subsequent metallization step determines the electrical conductor. This expands the accessible material space but also adds a downstream chemical step that must be included when assessing throughput, yield, and environmental burden. Its process-level material and waste comparisons should be distinguished from a complete-tag LCA, and its reported tag function does not by itself establish repeated production yield.

Finally, Svanda et al. [42] provide a useful process constraint from a chipless-RFID perspective: even when screen printing with silver paste on polyester film and paper is mechanically feasible, the lower conductivity of printed metallization degrades the resonant RCS features relative to copper. This result provides an important counterexample to treating successful pattern formation as evidence of adequate RF manufacturing quality. The acceptable process window depends on the electromagnetic margin of the design: geometries with strong, well-separated resonant signatures can tolerate more conductor loss, whereas marginal resonances may become unusable even when the printed feature is geometrically intact. Although this is chipless rather than IC-based UHF RFID evidence, the transferable manufacturing lesson is that process suitability must be judged against the electrical margin required by the specific antenna architecture.

Across these studies, screen printing therefore has no single manufacturing advantage independent of the application. Its main strength is compatibility with viscous inks, relatively thick deposits, and diverse flexible substrates; its limiting mechanisms shift among substrate absorption, dielectric interface effects, thermal consolidation, adhesion, registration, and chip attachment. A practical selection rule is to favor screen printing when the required feature dimensions are moderate, and the substrate can accommodate the ink rheology and post-processing needed for adequate conductor continuity. Claims of scalability should then be supported by completed-tag repeatability after bonding, mechanical conditioning, and environmental exposure rather than by printing feasibility alone.

**Figure 5:**
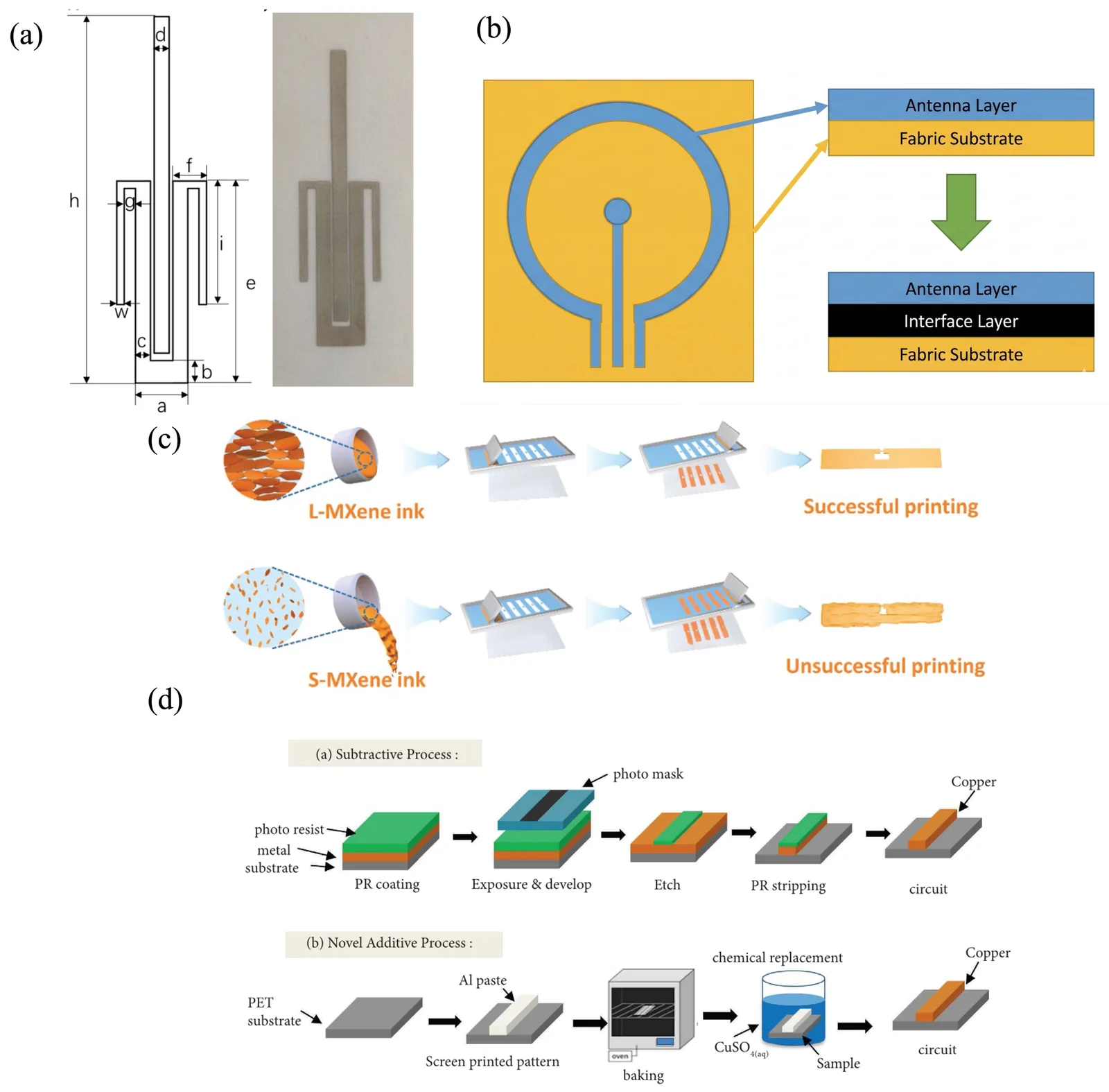
Screen-printing and substrate effects in flexible RFID antennas. (**a**) Geometry and textile-printed prototype of a UHF RFID tag antenna [33]. (**b**) Fabric antenna with an interface layer inserted between the conductive antenna layer and the textile substrate to reduce ink penetration and resonance shift [39]. (**c**) Schematic representation of the screen-printing workflow illustrating successful and defective pattern formation depending on ink rheology [44]. (**d**) Comparison between conventional subtractive fabrication and a hybrid additive route where an aluminum precursor is screen-printed on PET and subsequently converted into a copper conductive layer [43].

### 5.2. Inkjet Printing

Inkjet printing is a digital, maskless deposition process in which functional ink is delivered as discrete droplets, allowing conductive material to be distributed selectively across the antenna geometry. Its principal manufacturing advantage is therefore not simply pattern definition, but the ability to control local material allocation without modifying a physical mask or printing plate. This becomes particularly relevant in RFID antennas because the electrical importance of deposited conductor is strongly position-dependent: feed regions, impedance-sensitive features, and high-current paths generally impose tighter requirements on conductor continuity than electrically less critical portions of the layout. The trade-off is sensitivity to droplet breakup, substrate wetting, drying, and the continuity of successive layers. Consequently, geometric freedom is useful only when the ink–printhead–substrate combination preserves dimensional fidelity and sufficient electrical continuity after deposition and post-processing [45,46,47].

The inverse Ohnesorge number, $Z_{j}=\frac{\sqrt{\rho \gamma d_{n}}}{\eta }$, summarizes the balance among inertial, surface-tension, and viscous effects governing droplet formation, where ρ  is the ink density (kg/m^3^), γ  is the surface tension (N/m), $d_{n}\;$ is the characteristic nozzle diameter (m), and η  is the dynamic viscosity (Pa·s). Published $Z_{j}$ windows are empirical screening guides rather than sufficient conditions for satellite-free drop formation. Printhead geometry, driving waveform, ink rheology, and operating conditions also affect breakup. For manufacturing assessment, $Z_{j}$ should therefore be interpreted as a preliminary printability indicator rather than as a predictor of final conductor quality. An ink that falls within a nominal jetting window may still produce variations in line width, edge definition, or film continuity once droplet interaction with the substrate and successive deposited layers is considered. Drop formation should therefore be checked for the actual ink–printhead combination before selecting drop spacing and layer count [45,46,47].

The paired experiments of Oyeka et al. [48] provide a particularly useful example because they evaluate material placement within the same general fabrication and measurement framework rather than comparing unrelated antenna platforms. Oyeka et al. used the same 20 µm drop spacing and forearm mounting for a set of epidermal tags. Selective deposition of three additional layers around the feed, slot, and ports increased the threshold-derived range by up to 26%, whereas trimming low-current regions saved ink at the cost of range. The comparison indicates that ink usage and RF performance are not coupled simply through total deposited volume; the electromagnetic location of the additional conductor is equally important. Selective reinforcement is therefore most effective where current density, conductor loss, or matching sensitivity makes local resistance consequential, whereas material reduction is better directed toward regions with lower electrical sensitivity.

Marindra et al. [31] provide the complementary case by removing conductor rather than selectively adding it. Removing conductive material from the interior of the meander arms reduced the reported ink volume from 0.428 to 0.230 mm^3^, corresponding to an approximately 46% reduction, while the threshold-derived range decreased from 5.54 to 4.71 m, or approximately 15%. These are measurement-based range estimates, not a field demonstration over those distances. The result establishes a quantifiable material–link-margin trade-off: an approximately 46% reduction in ink consumption was accompanied by only an approximately 15% reduction in the threshold-derived range. Whether this trade-off is acceptable therefore depends on both the minimum reading distance and the manufacturing objective. If material consumption is a primary constraint and 4.71 m remains above the required operating distance with sufficient deployment margin, the reduced-ink geometry provides a meaningful manufacturing benefit. Conversely, when maximum range or tolerance to environmental detuning is prioritized, the lost link margin becomes more consequential.

The modeled range reduction was smaller, which also indicates that nominal electromagnetic optimization alone may underrepresent fabrication-related penalties introduced by the realized conductor geometry. When interpreted together with Oyeka et al. [48], the two studies support a common inkjet design rule: conductor should be allocated according to local electromagnetic sensitivity rather than removed or added uniformly across the antenna.

Table 5 therefore separates direct UHF IC-tag evidence from supporting RF fabrication studies. Dielectric sensors [49], graphene RF structures [50], and printed waveguide or array implementations [51] demonstrate material or integration possibilities, but their reflection coefficient and bandwidth are not UHF IC-tag read-range measurements. Their value in this review lies instead in identifying manufacturing capabilities—such as multilayer deposition, substrate preparation, conductor consolidation, and dielectric integration—that may be transferable to RFID fabrication when subsequently validated at complete-tag level.

Distributed printed RF structures provide a second type of supporting evidence because they clarify how inkjet-defined conductors interact with multilayer dielectric geometry. Anelli et al. combined inkjet-patterned PET sheets with 3D-printed PETG layers to form a partially reflecting surface for a Fabry–Pérot antenna; the relevant manufacturing lesson is that dimensional control of the printed conductor cannot be separated from control of the dielectric spacing when the electromagnetic response depends on a multilayer distributed structure [52].

Jaiswar et al. represented carbon-nanotube frequency-selective surfaces and dielectric layers with an electrical equivalent circuit and adjusted the number of printed layers to control resistive loading and absorption [53]. This example is relevant because it links repeated deposition directly to an effective electrical parameter: increasing the number of printed layers modifies resistive loading rather than merely increasing nominal conductor thickness. However, the equivalent-circuit representation describes the distributed electromagnetic response of the printed structure; it is not evidence of a physically printed lumped matching network. For UHF tags, the transferable principle is to connect trace/gap geometry, film resistance and substrate loading to the antenna input impedance, then verify chip power transfer and radiation efficiency experimentally. Microwave absorber or Fabry–Pérot antenna results cannot be treated as evidence of UHF RFID read range.

Taken together, the reviewed studies position inkjet printing most strongly where digital pattern modification, selective conductor placement, and low-tooling fabrication are more valuable than intrinsically thick single-pass deposition. Its principal limitations arise from the coupled control of droplet formation, wetting, layer-to-layer continuity, dimensional fidelity, and post-deposition conductor consolidation. For UHF RFID, the most informative evidence therefore comes from paired designs fabricated and tested under common conditions, because such comparisons reveal whether material savings or local reinforcement produce a genuine manufacturing advantage rather than an apparent improvement caused by different chips, substrates, or measurement conditions. Process scalability should ultimately be assessed from the stability of these properties across fabricated tags, not from nominal printability alone.

**Table 5:** Inkjet and related RF fabrication studies with explicit scope.

Device/Study	Substrate	Conductor/Dry Film	Processing	Geometry/Frequency	Validation/Scope
Epidermal UHF [48]	Epidermal platform; carrier material NR in this comparison	Sun Chemical Ag nanoparticles; film thickness NR	20 µm drop spacing; two full layers plus one to three selective layers; curing NR	Grid tracks: 0.2 or 1 mm; 865–868 MHz	Tagformance measurements on the forearm; paired relative comparison
Meander UHF [31]	PET, 135 µm; εr = 3 and tanδ = 0.002 as design inputs	Mitsubishi NBSIJ-MU01 Ag; ≈1 µm film is a datasheet expectation	Brother MFC-J825DW; curing NR	0.25λ meander; comparison at 922.5 MHz	Threshold-based estimates: 5.54 to 4.71 m; ≈46% less ink; threshold-derived range 5.54 to 4.71 m (≈15% reduction)
Fruit UHF [54]	PET, 0.2 mm	Ink chemistry and film thickness NR	Curing NR	902–928 MHz operating band	Controlled and practical fruit tests must be distinguished
Microwave loop comparator [49]	Kapton, 0.19 mm; εr = 3.5, tanδ = 0.0027	Ink chemistry NR; design-table conductor thickness 0.008 mm	50 °C, 15 min	50 × 70 mm; nominal 2.4 GHz sensor	RF resonator; Voltera NOVA deposition mode needs clarification before use as a droplet-process benchmark
Reactive graphene comparator [50]	Plasma-treated PET	Aqueous GO and 45% HI; repeated layers	70 °C platform; overnight vacuum drying at room temperature	Dipole arms: 75 × 5 mm each; measured resonance 864 MHz	RF antenna demonstration; no chip/read-range validation
SIW/array comparator [51]	RO4003C, PI and PEN	Ag nanoparticles; reported conductivity 9.1 × 10^6^ S/m; conductor 15 µm	Stepped 40–200 °C; SU-8 bonding: 120 °C, 20 min	5.8 GHz SIW; 27.5–28.5 GHz array	Supporting multilayer and via-fabrication evidence

### 5.3. Aerosol Jet Printing

Aerosol jet printing (AJP) atomizes ink and focuses the aerosol with a sheath gas for non-contact deposition. Fine features, local multilayer build-up, and compatibility with non-planar surfaces make it useful for printed RF prototyping, while line quality remains sensitive to gas flow, substrate temperature, and post-processing [55,56,57]. Compared with screen printing and conventional inkjet deposition, its principal manufacturing value lies in localized, non-contact material delivery where geometric fidelity, access to three-dimensional surfaces, or selective thickness build-up is more important than maximum areal throughput. These advantages, however, depend on maintaining a stable deposition window because aerosol focusing, overspray, substrate interaction, and thermal consolidation jointly determine the final line geometry and electrical continuity [58,59].

Its relevance to RFID is greatest when fine feed gaps, selective material placement, or unconventional substrates are required. Accordingly, demonstration of narrow or conformal features should be interpreted as evidence of deposition capability rather than, by itself, as evidence of scalable tag manufacture. For RFID production, geometric precision must ultimately be accompanied by repeatable conductor resistance, compatible thermal processing, reliable chip interconnection, and acceptable device-to-device variation.

Verma et al. [60] illustrate this distinction particularly well. Their five HF RFID antennas establish process feasibility on fiber-based paper, but the observed device-to-device variation also defines the current evidentiary limit of the demonstration: three repeatedly communicated with the reader, one produced an unreliable response, and one did not communicate. The case-study antennas were cured at 120 °C for 180 min; the separate line-screening experiments used other thermal settings. The significance of these results is therefore not a production-yield estimate, but evidence that successful AJP pattern formation does not necessarily translate into uniform assembled-device functionality. The combination of long thermal treatment and variable series resistance indicates that curing history and conductor consolidation remain integral parts of the process window, particularly on thermally and morphologically sensitive paper substrates.

A related coated-paper HF label combined the antenna with an interdigital sensor and a polyaniline layer [60]. This study extends the relevance of AJP from conductor deposition to functional co-integration, where several printed elements must coexist on the same substrate without compromising the RF interface. Read together with Verma et al. [60], it also shows that substrate preparation and post-deposition thermal treatment are not secondary finishing operations: they directly condition conductor continuity, interfacial quality, and the compatibility of subsequently integrated functional layers. This is evidence of functional integration by AJP, not a UHF read-range benchmark.

For UHF RFID, Chietera et al. [27] provide a more direct within-study comparison of conductor realization. An AJP silver structure and a conductive-filament structure were fabricated on three-dimensional supports and characterized through activation sensitivity. Their estimated maximum ranges were approximately 7 m and 3 m, respectively. Because both implementations were evaluated within the same general antenna architecture, the difference provides stronger evidence of the influence of conductor realization than a comparison of read ranges drawn from unrelated studies. The AJP implementation benefited from the lower-loss metallic conductor and finer deposited geometry, but the result should remain specific to that construction. The printed ABS support and commercial coupling loop distinguish it from a thin, fully printed flexible label; independent sample statistics and continuous production were not established. Thus, the study supports AJP when conductor quality and geometric control dominate the design requirement, while offering limited evidence regarding web-scale throughput or complete flexible-label manufacturing.

Wiklund et al. [61] address a different criterion that is often omitted from process comparisons: whether the printed structure preserves its electrical and RF properties after environmental exposure. They printed coplanar waveguide-fed slot antennas on acetylated cellulose nanofiber substrates and compared them with PET references before and after accelerated photodegradation (Figure 6c). Although the antenna was designed for 3.5 GHz rather than UHF RFID, the comparison is valuable because it separates initial electromagnetic performance from performance retention over time. The antennas printed on ACNF showed somewhat lower initial realized gain than those fabricated on PET, but after 1000 h of photodegradation they retained almost unchanged electrical behavior, whereas the PET-based devices exhibited coating peeling, a substantial increase in sheet resistance, and severe degradation in gain and efficiency. This comparison reveals an important process-selection trade-off: the substrate producing the strongest initial RF response is not necessarily the one that provides the most stable conductor–substrate interface after aging. For printed RFID manufacturing, durability evidence should therefore complement initial sheet resistance and RF measurements whenever the intended service environment includes prolonged light, thermal, or environmental exposure. The result supports ACNF as a durability-relevant substrate within the tested AJP configuration; it should not be generalized into a broader environmental or life-cycle advantage without corresponding assessment evidence.

A transparent silver-mesh antenna integrated with a solar cell [62] supplies a further AJP fabrication example (Figure 6d). Its relevance is primarily integrative rather than RFID-specific: the study demonstrates that AJP can define sparse metallic patterns while preserving optical transmission and compatibility with an adjacent energy-harvesting component. Such multifunctional integration is relevant when antenna fabrication must accommodate competing optical, electrical, or packaging requirements, but the associated microwave results do not establish passive UHF RFID read range, chip integration, or manufacturing yield.

**Figure 6:**
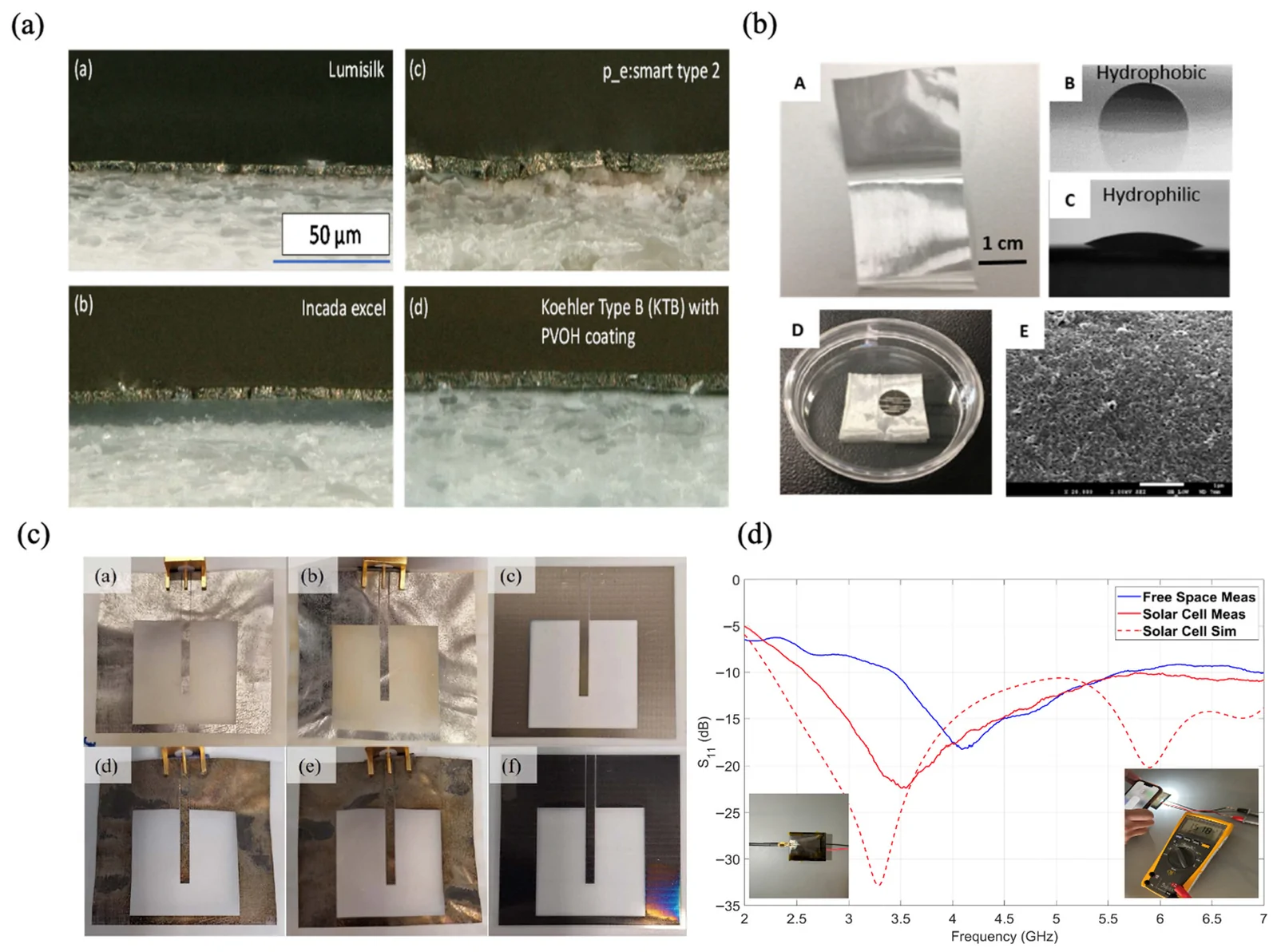
Representative examples of aerosol jet printed antenna platforms and substrate-related considerations. (**a**) Cross-sectional optical images of AgNP patterns printed on different paper substrates, highlighting differences in coating continuity and substrate compatibility [60]. (**b**) Biodegradable scaffold-based antenna platform, including scaffold morphology, wettability modification, printed antenna geometry, and surface microstructure [63]. (**c**) Antennas printed on acetylated cellulose nanofiber and PET substrates before and after photodegradation [61]. (**d**) Transparent antenna integrated with a photovoltaic cell, together with the measured and simulated reflection coefficient under free-space and integrated conditions [62].

Taken together, these studies position AJP as a process selected primarily for geometric precision, localized deposition, conformal access, and functional integration rather than for demonstrated high-volume RFID throughput. The principal manufacturing trade-off is therefore not simply precision versus process time, but the value of that precision relative to the additional requirements for aerosol stability, substrate preparation, thermal consolidation, and reproducibility. AJP is particularly appropriate when fine feed regions, three-dimensional supports, selective multilayer build-up, or heterogeneous functional integration provide a clear electromagnetic or architectural benefit. By contrast, when the antenna geometry is tolerant of coarser features and the dominant requirement is high-volume production, the added process complexity of AJP offers a less compelling advantage. Current evidence is strongest at the level of fabrication capability and application-specific prototypes; scalable UHF RFID manufacture would require broader evidence on repeated complete tags, chip attachment, process stability over extended runs, and post-conditioning RF performance.

### 5.4. Roll-to-Roll Printing Techniques: Flexography and Gravure

Roll-to-roll describes a continuous substrate-handling format; flexography and gravure describe ink-transfer mechanisms. Screen and inkjet printing can also be configured for continuous processing. Consequently, a laboratory demonstration of a printing mechanism is not evidence of web-scale production. For manufacturing assessment, nominal printing speed should therefore be interpreted together with run length, conductor uniformity, registration accuracy, thermal processing, downstream conversion, and functional-device reproducibility. HF/NFC and chipless examples are used here only to assess transferable manufacturing constraints.

Flexography meters ink through a relief plate, while gravure transfers ink from engraved cells. Both can operate within a continuous web line, but feature formation, ink loading, and drying depend on the specific tool and substrate. Their manufacturing advantage is consequently conditional rather than intrinsic: high web speed is beneficial only when ink transfer preserves the conductor thickness, continuity, and dimensional fidelity required by the RF design. The appropriate comparison is the demonstrated process window, not an assumed universal resolution or speed advantage [64,65,66,67].

Wang et al. [68] provide the most direct UHF evidence among the flexographic studies considered here. Their R2R process printed a catalytic precursor pattern on paper, followed by electroless copper deposition to form the final RFID antenna conductor. The resulting copper tracks reached widths of approximately 89 µm and a resistivity of 2.22 µΩ·cm, while the completed tags retained approximately 96% of their initial reading distance after 800 bending cycles. These results demonstrate that flexography can support fine antenna pattern definition and mechanically robust UHF tag operation, but they also show that the final electrical performance depends on a hybrid manufacturing sequence in which electroless copper deposition is essential to conductor formation. Accordingly, the flexographic printing stage should be evaluated together with the subsequent metallization step when assessing total process complexity, throughput, and scalability.

Shrestha et al. [64] evaluated flexographic chipless patterns on a benchtop Flexiproof system at nominal printing speeds of 20, 40, 50, and 80 m/min. Print quality at 80 m/min was insufficient, and the reported comparisons focused on 20 and 50 m/min. Lower speed increased deposited thickness and reduced sheet resistance, improving the resonant response. Because the printing method, ink, and RF structure were maintained within the same experiment, this comparison provides a useful direct relationship between printing rate and conductor formation. Increasing speed reduced the transferred conductive material and degraded the electrical response, indicating that throughput cannot be increased independently of film quality. For flexography, the relevant operating limit is therefore the highest printing rate at which transferred ink volume, sheet resistance, and RF response remain within the required device tolerance. These results do not establish long-run R2R yield or passive UHF IC-tag performance.

Lepak-Kuc et al. [65] printed shrinkable PET using a sheet-fed flexographic press at 10 m/min. This example separates high-rate flexographic patterning from demonstrated continuous R2R manufacture. The subsequent shrinking operation altered the conductive layer, showing that conversion, package conformity, and electrical continuity must be evaluated after the final thermal and mechanical process rather than only on the flat printed film. For packaging-oriented RFID, this distinction is particularly important because the electrically relevant structure is the converted antenna after shrinkage and integration, not merely the conductor immediately after printing. Post-print transformation therefore becomes part of the manufacturability window.

For gravure printing, Kang et al. [69] provide an antenna-specific R2R benchmark in which the 13.56 MHz Ag antenna was continuously printed directly on a 75 µm PET web. The process operated at 8 m/min and was followed by continuous curing at 150 °C for 5 s; the optimized printed antenna reached a Q factor of approximately 13. Although the operating regime is HF/NFC rather than UHF, the study is valuable here because web speed, substrate, conductor deposition, continuous thermal treatment, and antenna electrical quality are reported for the same R2R-fabricated structure. The result therefore provides direct antenna-manufacturing evidence rather than using the yield or thickness of an unrelated printed component as a surrogate for antenna performance.

The same study connects multilayer registration to functional output, reported an overlay-registration error of approximately ±40 µm and indicated that approximately 80% of the printed rectennas supplied the required 3 mW under the reported loading conditions. The authors attributed the limited yield to overlay-registration accuracy. This result concerns the antenna–rectifier structure and its power-delivery criterion, identifying multilayer alignment as a manufacturing control point for preserving functional reproducibility [69].

Maskey et al. [66] illustrate a complementary gravure-based route involving subsequent silicon-chip integration. Their R2R gravure platform continuously fabricated an NFC antenna and thermistor as part of a time–temperature-history food label, after which the printed structure was interfaced with a silicon-chip transponder and other system components. This construction highlights the additional manufacturing requirements associated with converting printed RF components into a chip-integrated wireless label. Semiconductor attachment and integration of the remaining components introduce processing times and interconnection requirements that must enter the assessment of complete-label throughput.

They reported a gravure printing speed of 6 m/min on a 75 µm PET web, with a 150 °C in-line heating stage and a separate 250 °C post-print sintering treatment. These conditions illustrate a central limitation in interpreting R2R throughput: the nominal web velocity describes material transport through the printing unit, whereas the effective manufacturing rate also depends on the thermal history required to establish sufficient conductor quality. A production-speed comparison should therefore account for both continuous deposition and subsequent processing rather than treating web speed as a stand-alone scalability metric.

The influence of these downstream operations becomes quantifiable when printing and assembly are documented over defined production lengths. As a complementary R2R manufacturing benchmark, Hakola et al. used rotary screen printing to produce more than 2000 chip-free NFC–electrochromic label structures over an 80 m run on paper. Eleven layers were deposited in separate printing passes at speeds of 2–4 m/min. Thermal pretreatment improved substrate dimensional stability, while the silver and carbon layers required additional drying passes at 140 °C. These conditions show how multilayer registration and conductor consolidation add processing time to the nominal deposition operation. Subsequent chip assembly proceeded in an indexed, stop-and-go mode at a reported throughput of 360 labels per hour, with adhesive dispensing, including fiducial checks, setting the cycle time. In this workflow, increasing printing speed would therefore offer limited improvement in completed-label throughput while dispensing remained the assembly bottleneck.

Hakola et al. also provide a basis for distinguishing production volume from stage-specific functional yield. The assembly stage covered approximately 50 m and 1428 labels, of which 50 failed NFC detection, corresponding to a reported 96.5% bonding-stage yield. This percentage characterizes wireless communication after chip attachment within the assembled population; the 80 m printing run and the approximately 50 m assembly run represent separately reported production quantities. The interconnection process also exposed a concrete alignment–throughput trade-off: adhesive dots spread from approximately 200 µm to 350–400 µm during component placement, while fiducial checks consumed a substantial fraction of the dispensing time. Reducing those checks could accelerate assembly but increase the risk of misplaced adhesive. For printed UHF tags, the transferable design implication is to specify pad separation, adhesive volume, and placement verification together, and to evaluate their effects through the rate and yield of electrically functional interconnections.

Taken together, the studies identify different limiting operations within roll-based manufacturing. The flexographic examples emphasize ink transfer, conductor formation, and post-conversion continuity; the gravure examples connect multilayer alignment and thermal processing to RF function and subsequent integration; and the rotary-screen benchmark quantifies an assembly bottleneck and bonding-stage failures over a defined population. These observations support process selection according to the complete manufacturing sequence: flexography is attractive when rapid pattern transfer remains compatible with conductor formation and conversion, while gravure favors repeated production of stable layouts whose registration and curing requirements fit the web process. For either route, scalability is most meaningfully assessed through functional-label output per unit of production time, supported by actual operating speeds, processed lengths, registration measurements, and yield values tied to explicit test criteria and manufacturing stages.

**Table 6:** Cross-process evidence and conditional manufacturing choices.

Process/Representative Evidence	Demonstrated Feature Size	Dry-Film Thickness	Substrate Constraints/Post-Processing	Reported Printing or Web Rate	Reproducibility/Device-Level Evidence	Conditional Selection Rule
Screen printing [33,34,40]	≈100 µm graphene line [40]; Ag feature size NR [34]	Ag: 3.4 ± 0.2 µm [34]; graphene deposited in three passes [40]	Textiles/PI; Ag: 120 °C/30 min [34]; graphene: drying after each pass, annealing up to 320 °C/36 min plus compression [40]	170 mm/s squeegee speed [33,34,40]	Repeated electrical tests [33]; chip-bonding study [34]	Prefer for thicker deposits and viscous inks; substrate and chip-interface compatibility are limiting.
Inkjet Printing [31,48]	0.2 or 1 mm grid tracks [48]; 20 µm drop spacing is not line width	≈1 µm Ag in [31], manufacturer-based expectation	PET/epidermal platforms; wetting, drop coalescence, film continuity, and curing control conductor formation	Production rate NR	Selective reinforcement increased threshold-derived range by up to 26% [48]; ≈46% ink reduction accompanied ≈15% lower threshold-derived range [31]	Prefer for digital patterning and selective conductor placement.
Aerosol jet [27,60]	≈130–140 µm line in process screening [60]	Final antenna NR; [27] reports an 18 µm reference film on glass	Coated paper [60] and 3D ABS [27]; substrate morphology and thermal consolidation are critical; HF antenna: 120 °C/180 min [27]	15 mm/s coupon traverse speed [27]	3/5 antennas repeatedly communicated in [27]; Ref. [60] provides UHF prototype evidence but no production statistics.	Prefer when localized fine features or non-planar deposition provide a clear integration benefit.
Flexography [64,65,68]	≈89 µm Cu track [68]; feature size NR [64]	2.2/1.9 µm at 20/50 m/min [64]	Paper with electroless Cu metallization [68]; PET: 140 °C/20 min [64]; shrink conversion evaluated [65]	20/50 m/min in [64]; inadequate print quality at 80 m/min; sheet-fed 10 m/min [65]	UHF tags in [68] retained ≈96% of initial reading distance after 800 bending cycles; five-sample means [64]	Prefer for higher-rate pattern transfer when ink transfer and post-conversion continuity remain stable.
Gravure R2R [66,69]	≈1 mm antenna line with 0.5 mm spacing [69]; feature size NR [66]	Antenna dry-film thickness NR on a directly comparable basis	75 µm PET, 150 °C/5 s continuous curing [69]; 75 µm PI, in-line heating plus post-print sintering [66]	8 m/min [69]; 6 m/min [66]	≈80% functional rectenna yield; overlay-registration error ≈±40 µm; >90% yield for the laminated wireless QR-code system [69].	Prefer for repeated high-volume web production with stable geometry, curing, and registration.

## 6. System-Level Validation and a Focused Computational Outlook

System-level validation asks whether a completed tag can be activated and decoded under stated conditions, including after mechanical or environmental exposure. Simulated impedance, a printed antenna without an attached IC, a threshold-based range estimate, and a distance-tested tag answer different questions. Table 7 separates these forms of evidence and reports missing test conditions explicitly.

### 6.1. Definitions and Minimum Reporting Requirements

A directly measured read distance is a physical reader–tag separation at which a specified response was observed. A threshold-based range estimate is extrapolated from a measured activation threshold at a known distance, often using a calibrated free-space path and an assumed EIRP. A simulation-based range is calculated using modeled gain, matching, and chip sensitivity. “Theoretical maximum” denotes the limit of that model; it is not a field observation. “Read range” is the umbrella term used here only when the evidence type is stated. Reading an identifier and retrieving a valid sensor value may have different power requirements [31,54,70].

A reproducible report should state frequency and channel, reader model, EIRP and calibration plane, chip identity and activation sensitivity, polarization, antenna orientation, mounting surface and spacer geometry, propagation environment, and the physical test distance. It should also specify the success criterion, number of tags and repeated trials, variability, and the mechanical state. For sensing, the accuracy and usable sensing distance should be reported separately from identifier readability.

### 6.2. Evidence Levels and Cross-Study Comparability

Evidence is recorded along separate dimensions: simulation only; fabricated prototype; controlled RF measurement; repeated specimens or trials; bending or washing; and application or field testing. These are not interchangeable ranks. For example, a single outdoor demonstration may have greater environmental relevance but less quantified repeatability than a controlled multi-sample experiment. A durability test on a microwave antenna supports a substrate/process claim, not a UHF tag read-range claim.

Within-study comparisons provide the clearest basis for attributing performance differences because the chip, reader configuration, calibration, and test protocol are held constant while a specific design or manufacturing variable is changed. As discussed in Section 2.1, cross-study read-range values remain strongly dependent on the complete link conditions. Differences in EIRP, chip sensitivity, polarization, mounting environment, and reverse-link constraints can therefore prevent direct comparison, even when a simple power normalization is applied. Reported ranges should consequently be interpreted within their original test conditions.

### 6.3. Computational Methods for Printed-Tag Variability

In this review, computational methods are retained only where they can incorporate the fabrication, mounting, and environmental variability summarized in Table 7 into tag design or performance interpretation. Impedance optimization can balance chip activation and backscatter modulation rather than minimizing a single reflection coefficient [17,71,72]. Beyond impedance-level optimization, geometry-search methods can explore manufacturable design variables such as trace widths, gaps, and substrate dimensions. The simulation-based PSO study [71] provides one example of this approach. However, such optimization does not establish robustness to production variation unless those variations and independently fabricated tags are included in the validation.

Reader deployment and localization studies show that geometry of interrogation and the signal model influence measured response [73]. For printed tags, their transferable value is whether calibration or inference remains stable after deformation, moisture-induced detuning, mounting changes, or batch-to-batch variation [72,73,74]. The cited system studies do not establish that capability for printed-tag production. A useful next step is to train and test a tolerance-aware model on independently fabricated batches, with separate mounting and aging conditions, and compare its predictions with measured thresholds and decoding reliability.

Dual-interface UHF/HF sensors illustrate a further distinction: communication capability does not establish sensing performance at the same distance. Such architectures remain supporting system examples unless their printed-tag construction and required function are validated together [75]. Harmonic communication and object-centric data management are outside the principal manufacturing comparison [76,77]. Accordingly, the computational outlook is focused on tolerance-aware design and validation of printed tags rather than on general RFID system optimization.

## 7. Environmental Sustainability and Circularity of Printed RFID Tags

Material substitution alone provides limited evidence of environmental improvement. Paper-based substrates, reduced ink consumption, and lower curing temperatures may offer favorable environmental attributes, but their overall benefit depends on conductor production, chip and adhesive content, encapsulation, service life, and end-of-life treatment [20,78,79]. Accordingly, this section distinguishes quantitative life-cycle assessment (LCA) results from material- and process-level sustainability indicators and relates both to demonstrated device functionality.

Complementary evidence addresses the end-of-life burden associated with large-scale RFID deployment rather than product-level life-cycle impact. Bukova et al. [80] modeled RFID-related electronic waste entering municipal waste streams in Slovak households, illustrating how high tag consumption can translate into cumulative end-of-life material flows. In a subsequent logistics-center case study, Bukova et al. [81] estimated approximately 5.7 t/year of RFID-related electronic waste associated with identifiers on received pallets, including approximately 139 kg of metal. These studies provide deployment-scale evidence of waste generation and reinforce the relevance of collection, reuse, and recycling strategies; their outcomes should, however, be interpreted separately from comparative tag-level LCA because they quantify waste flows rather than complete life-cycle impacts.

Environmental evidence must be classified by assessment boundary and functional unit. Aliakbarian et al. [78] compared one standard plastic-based UHF tag with one paper-based tag using an attributional LCA covering materials, manufacturing, packaging, transport and end of life; use-phase impacts were explicitly excluded. Their reported GWP values were 0.0152 and 0.0118 kg CO_2_-eq per tag, respectively, with an approximately 23% reduction reported for the paper configuration. This is a comparison of two architectures with different masses and inventories, not a universal environmental advantage of paper. Table 8 separates this result from prospective models and recycling experiments (Figure 7a).

Ding et al. [82] illustrate why these boundaries matter. Their current and emerging component scenarios use a functional unit of producing one tag or reader and a cradle-to-gate boundary. Their battery supply-chain scenarios instead include tags, readers and backend servers per 1 kg of battery module produced and delivered, using a cradle-to-site boundary. In contrast to the configuration-specific reduction reported by Aliakbarian et al. [78], Ding et al. [82] found no significant difference between representative current paper- and PET-based tags across the four impact indicators emphasized in their main analysis. The prospective 25–70% reductions for emerging tags and approximately 3–5% net reductions for modeled battery supply chains depend on material, energy and demand assumptions; they are not measured savings for flexible printed packaging labels (Figure 7b).

The NFC gas-sensor case of Zikulnig et al. [79] provides supporting manufacturing evidence rather than UHF performance evidence. Its functional unit is one sensor tag operating for one day, and its streamlined assessment excludes transport and use/readout energy. Changing substrate, conductor, printing method and end-of-life scenario together reduced modeled GWP from 42 to 25.7 g CO_2_-eq per tag. However, the study also observed cracks interrupting copper traces on bio-PE and assessed recyclability of individual materials rather than demonstrating recycling of the complete product. The minimum modeled footprint therefore requires independent confirmation of functional equivalence and durability.

Recycling experiments address a different question. Pogačar et al. [20] evaluated RFID-printed label paper using INGEDE 11 deinking and recycled-handsheet measurements. Ink removal was feasible, but residual dirt-particle counts and contaminated area increased in RFID-containing samples. This evidence informs compatibility with paper recycling; it does not quantify a full-life-cycle benefit or establish harmless disposal of conductive residues (Figure 7c).

Lower conductor mass, lower curing temperature, renewable feedstocks and degradability are design indicators that may motivate a comparative assessment. They are not sufficient evidence of lower total environmental impact. Such an assessment must include ink synthesis and additives, drying or sintering energy, chip and adhesive burdens, encapsulation, service life, replacement frequency and the actual collection and treatment route. A lighter tag that fails prematurely or needs additional protective layers can shift burdens elsewhere in the product system [78,79,82].

Durability and reuse must be distinguished from environmental impact. Hakola et al. [83] studied printed NFC tags and protected commercial UHF tags under washing and heating, showing that the adhesive and protective construction affect survival. The device classes and protocols must remain separate; the commercial UHF results do not validate the durability of a newly printed UHF antenna (Figure 7d). A longer usable lifetime may reduce replacements, but the protective material and recycling consequences must also enter a comparative inventory.

Future comparisons should join RF and environmental inventories for the same tag architecture. Report conductor and chip mass, substrate and protective layers, process energy, independent specimen yield, functional lifetime and realistic end-of-life assumptions. Comparisons per manufactured tag are insufficient when read reliability or replacement rates differ; the functional unit should also describe the identification or sensing service delivered. Priority should be given to paired experiments that verify RF performance and mechanical endurance alongside transparent lifecycle scenarios, rather than inferring sustainability from substrate identity alone.

## 8. Conclusions and Outlook

This review identifies the coupling between electromagnetic design, conductor formation, and chip integration as a central engineering constraint in flexible printed passive UHF RFID tags. The strongest experimental conclusions arise from comparisons performed within individual studies under common fabrication and measurement conditions. These comparisons show that selective conductor reinforcement can improve activation performance, whereas reduced-ink layouts exchange material savings for lower RF margin. Substrate morphology influences the continuity of deposited conductors, while chip-bonding conditions affect contact quality and local track integrity. Together, these findings support allocating conductor according to electromagnetic sensitivity and evaluating material–process combinations through the electrical response of the completed tag rather than through nominal material properties alone.

Manufacturing evidence supports process selection according to the required conductor thickness, feature tolerance, substrate compatibility, and integration sequence. The reviewed processes provide different combinations of deposition thickness, geometric control, and continuous-processing capability, but their practical value depends on subsequent consolidation, metallization, registration, chip attachment, and final assembly. The engineering objective is therefore a reproducible manufacturing window that preserves antenna–chip power transfer and adequate radiation efficiency through final conversion. Evidence for this objective remains uneven: individual fabrication capabilities are comparatively well demonstrated, whereas sustained production of qualified UHF tags with documented device-to-device variability and complete-tag yield remains much less established.

The first research priority is reproducibility at the completed-tag level. Independent fabrication batches should establish distributions of activation threshold, impedance response, and decoding success under calibrated interrogation conditions, followed by mechanical and environmental conditioning representative of service. Directly measured distances, threshold-derived range estimates, and simulation-based predictions should remain distinct reporting categories. Connecting controlled laboratory measurements with application-level testing would then establish how fabrication variability, mounting conditions, deformation, and aging consume the available operating margin.

The next priority is to connect these performance distributions to manufacturing output. Extended production trials should quantify functional yield and throughput after conductor processing, chip attachment, interconnection, and protection, with failures traced to specific manufacturing operations. Environmental assessment should subsequently use the same validated tag architectures and an equivalent identification or sensing service as the functional basis for comparison. Existing life-cycle studies support configuration-specific conclusions, but broader interpretation requires inventories that incorporate production losses, protective materials, functional lifetime, replacement frequency, and realistic recovery or disposal routes. This connection would make reliability and manufacturing efficiency explicit determinants of environmental performance rather than treating sustainability as an independent material-selection criterion.

Tolerance-aware computational optimization, alternative conductors such as graphene and MXenes, and degradable material systems remain promising but comparatively less validated development directions. Their broader applicability requires verification across independently fabricated tags, relevant exposure conditions, and complete-device integration. Computational models particularly require validation against unseen fabrication batches and mounting conditions, while emerging material systems require joint assessment of RF stability, processing requirements, mechanical durability, and end-of-life behavior. Progress toward scalable and sustainable printed RFID should therefore be assessed through reproducible service over a defined lifetime, linking experimentally demonstrated RF performance to functional manufacturing yield and quantitatively supported environmental outcomes.

## Figures and Tables

**Figure 1:**
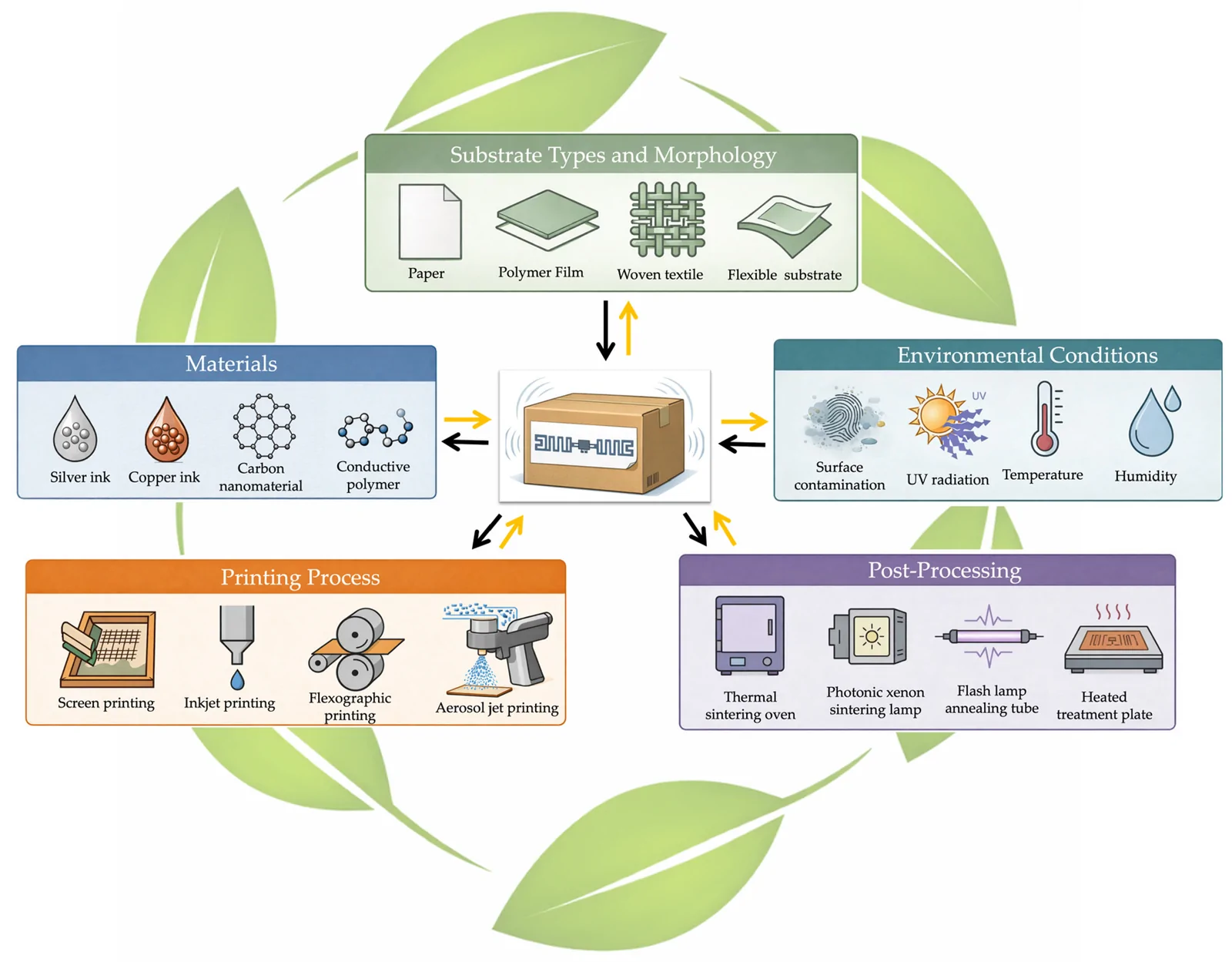
Key manufacturing and environmental factors influencing the performance of flexible printed UHF RFID antennas.

**Figure 2:**
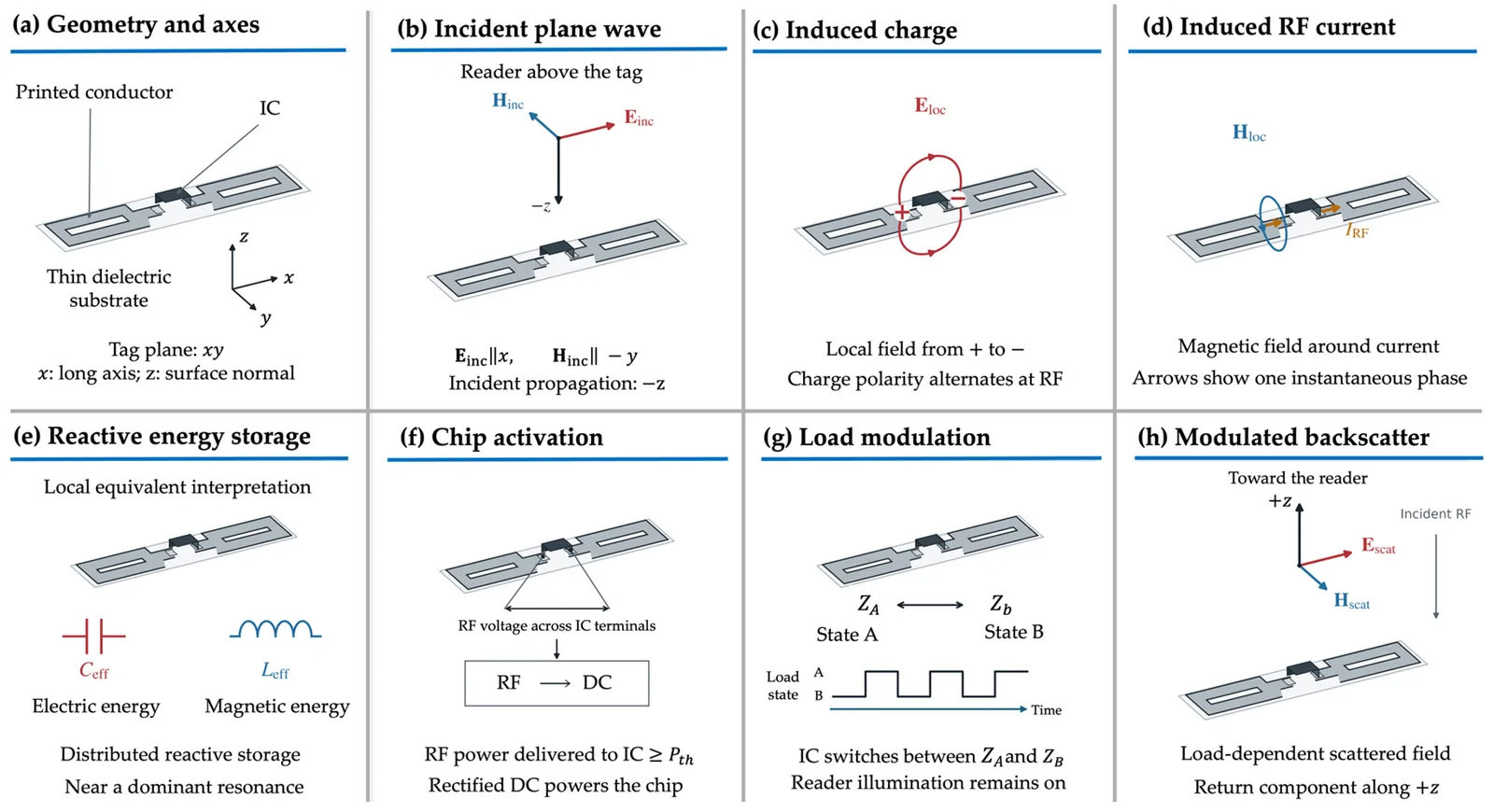
Electromagnetic and circuit-level operation of a passive UHF RFID tag. (**a**) Tag geometry and coordinate system. (**b**) Incident reader field propagating toward the tag. (**c**,**d**) Induced charge separation and RF current, together with their associated local electric and magnetic fields. (**e**) Equivalent-circuit interpretation of distributed reactive energy storage through $C_{eff}$ and $L_{eff}$ near resonance. (**f**) RF rectification and chip activation when the delivered power reaches the activation threshold $P_{th}$. (**g**) Load modulation through switching between the chip impedance states $Z_{A}$ and $Z_{B}$. (**h**) Resulting load-dependent modulated backscatter directed toward the reader. Field patterns and geometry are shown schematically and are not to scale.

**Figure 3:**
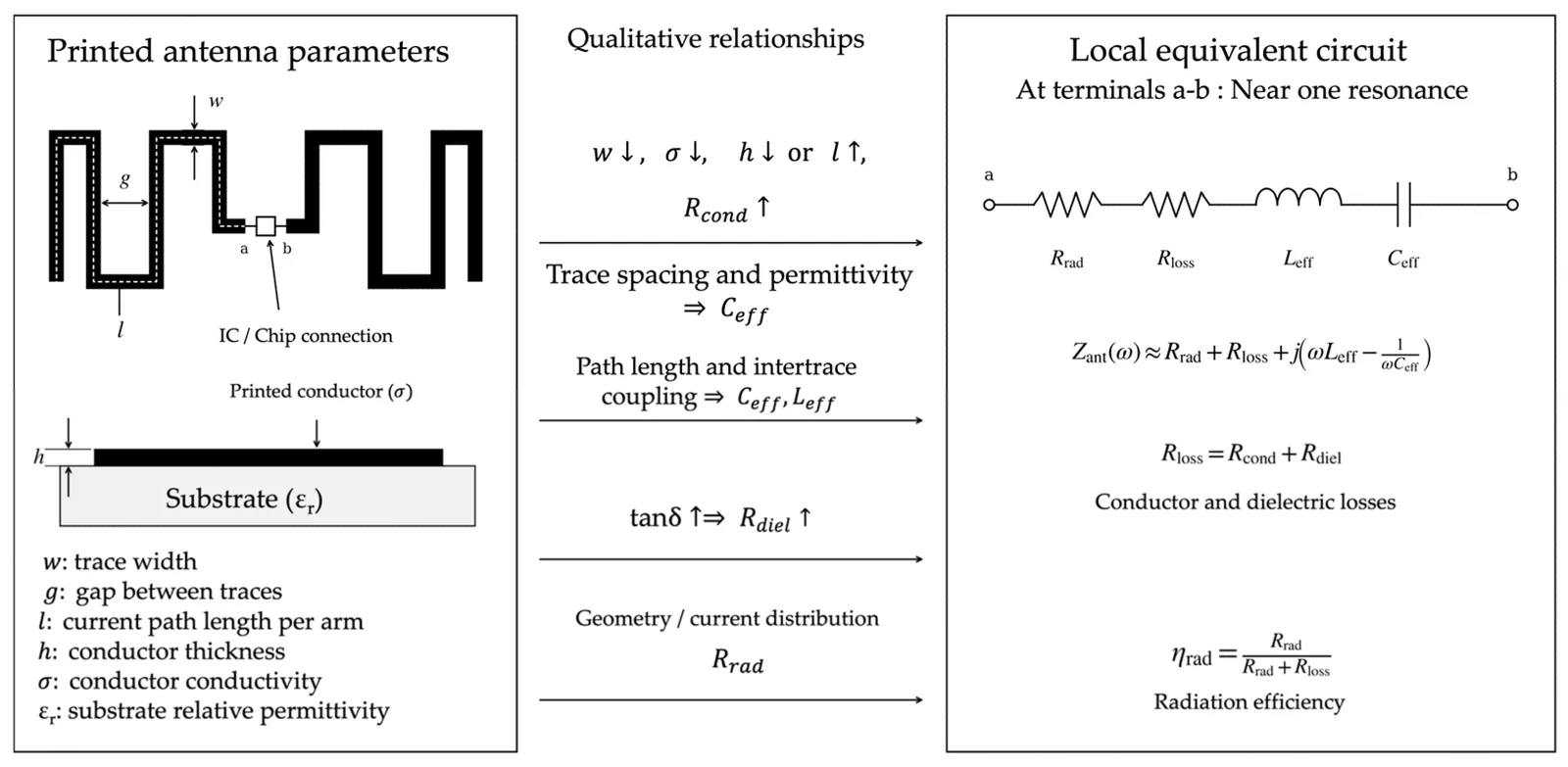
Representative local equivalent-circuit interpretation linking printed-antenna geometry and material parameters to impedance and radiation efficiency.

**Figure 4:**
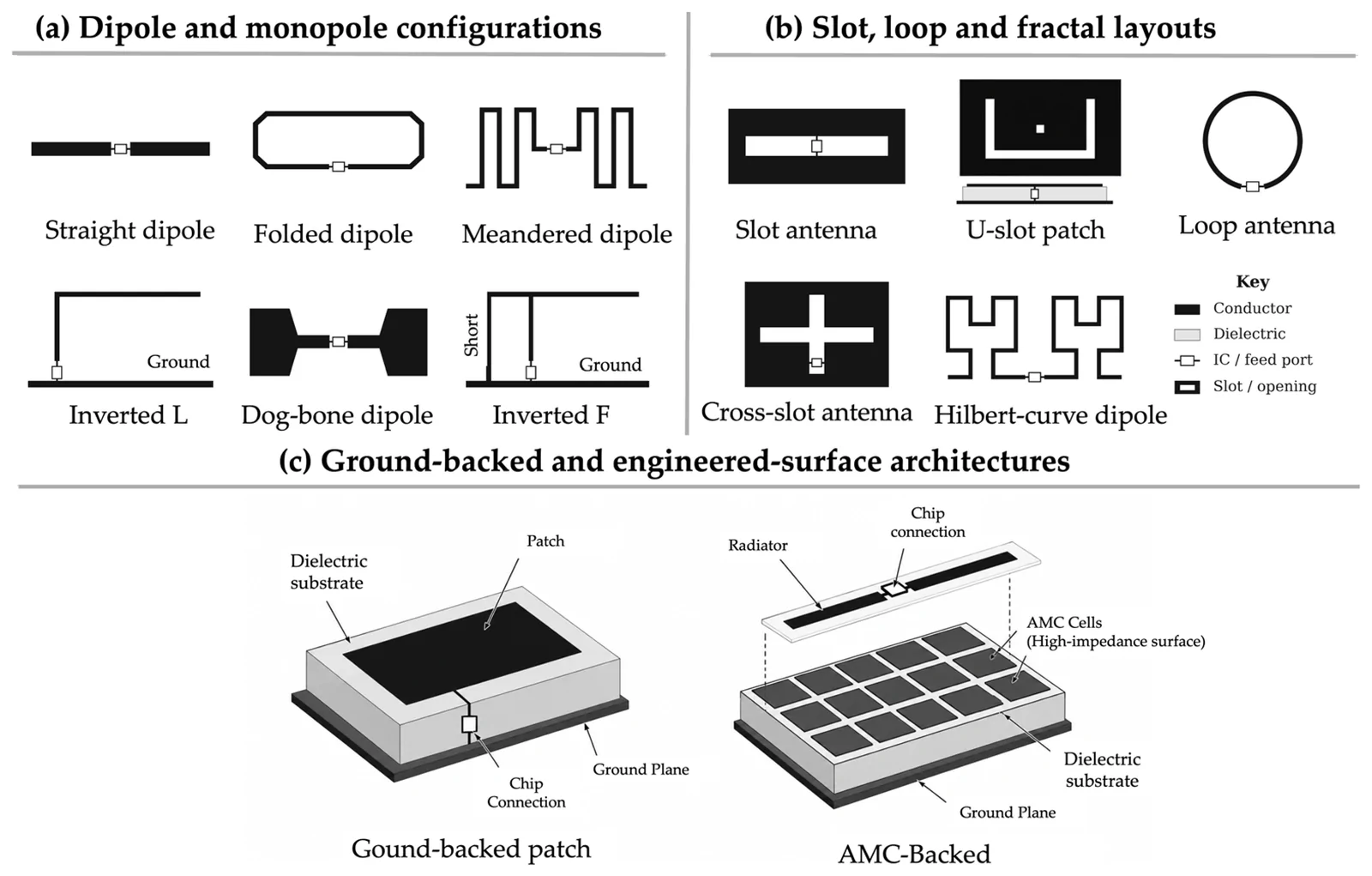
Geometry-driven design strategies for passive RFID tag antennas. Representative conceptual geometries used to control electrical length, impedance response, current distribution, and mounting tolerance. (**a**) Dipole- and monopole-derived configurations, including straight, folded, and meandered dipoles, inverted-L and inverted-F structures, and a dog-bone dipole. (**b**) Slot-, patch-, loop-, and fractal-based layouts, including slot and cross-slot antennas, a U-slot patch, a loop antenna, and a Hilbert-curve dipole, illustrating alternative approaches for shaping current paths and tuning the antenna response within compact footprints. (**c**) Platform-tolerant architectures represented by a ground-backed patch and an artificial magnetic conductor (AMC)-backed radiator, illustrating the use of ground planes and engineered surfaces to reduce interaction with the supporting platform. Geometries are schematic and not to scale.

**Figure 7:**
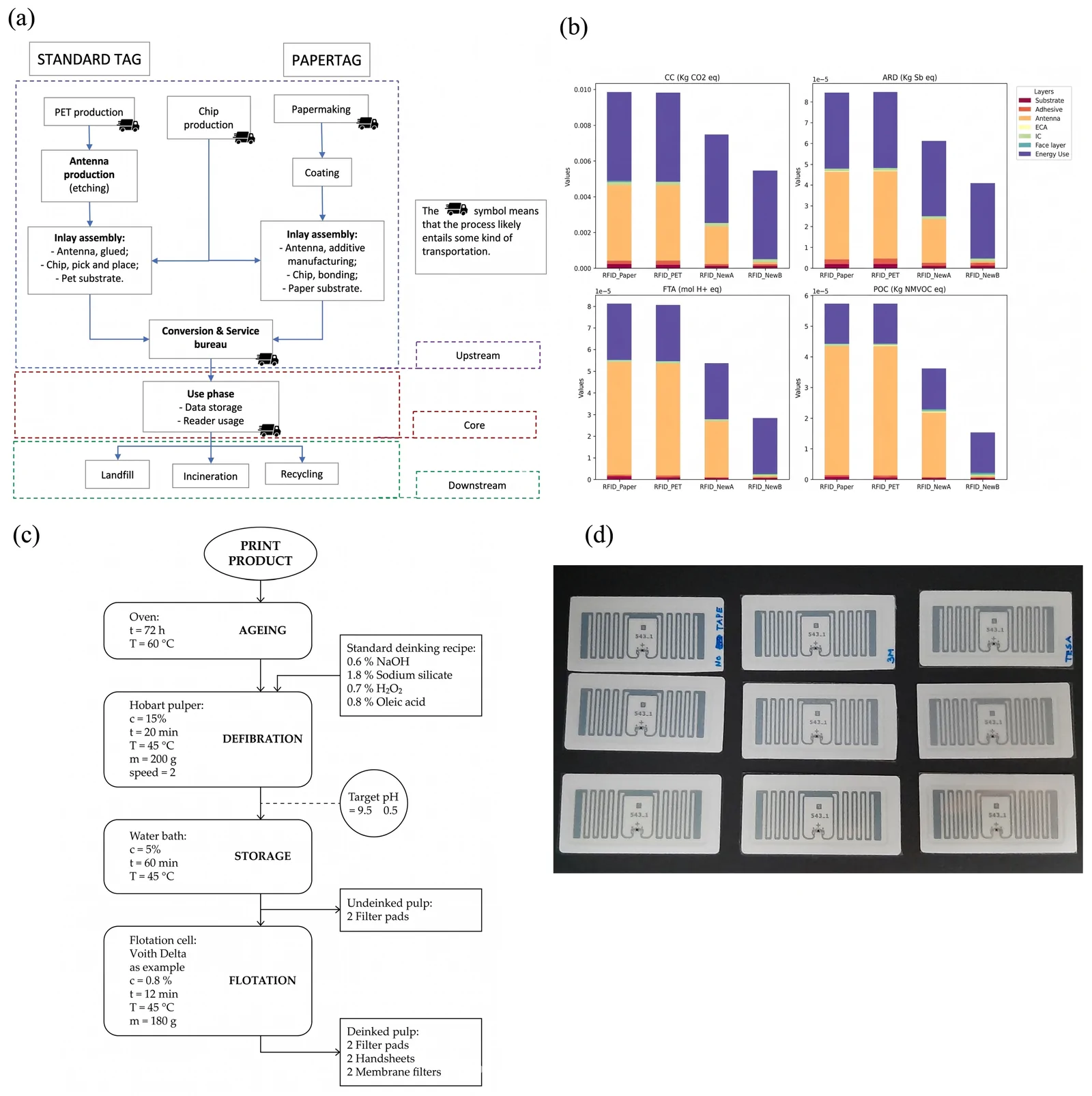
Selected examples illustrating environmental assessment, recycling compatibility, and durability of RFID tags. (**a**) Cradle-to-grave system boundaries considered for the comparison between a standard RFID tag and a paper-based RFID tag [78]. (**b**) Comparative environmental impacts of current and next-generation RFID tags in selected LCA categories [82]. (**c**) Experimental sequence used for the recyclability and ink-removal evaluation of RFID-printed paper samples following the INGEDE 11 protocol [20]. (**d**) Visual condition of protected commercial RFID tags after repeated washing and heating cycles, representing their durability under reusable packaging conditions [83].

**Table 1:** Comparison of the scope of closely related reviews and the contribution of the present synthesis.

Review	Scope and Emphasis	Extension Provided by the Present Review
Hamidon et al. (2024) [4]	Passive RFID antennas on flexible substrates for long-distance operation, with emphasis on conductivity, layer thickness, surface morphology, paper/polymer/textile substrates, inkjet printing, screen printing, and electroless plating.	Extends the process comparison to additional scalable printing routes, including aerosol jet, flexography, and gravure, under common manufacturing criteria, while incorporating complete-system validation and environmental evidence into process assessment.
Yang et al. (2023) [5]	Flexible RFID antenna design, conductive and substrate materials, fabrication technologies, and applications, including relationships among material selection, structural design, prototype fabrication, and performance measurement.	Examines the comparability of reported device-level results when printing conditions, complete-tag assembly, RF link conditions, measurement methods, and durability evidence differ across studies.
Wang et al. (2025) [8]	Printed RFID systems for sustainable IoT, including conductive inks, antenna architectures, scalable manufacturing, and broad material–architecture–process interactions involving inkjet, screen printing, direct ink writing, and intelligent or hybrid manufacturing routes.	Evaluates the strength of experimental validation, identifies which reported read ranges share sufficient test context for meaningful comparison, and distinguishes qualitative sustainability statements from conclusions supported by quantitative life-cycle evidence.
Present review	Flexible printed passive UHF RFID tags incorporating an integrated circuit (IC), with other frequency regimes included only as explicitly labeled supporting evidence.	Provides a condition-aware synthesis linking manufacturing variables to device performance, assessing the comparability of reported metrics under different test conditions, and evaluating sustainability claims within complete-tag and assessment-boundary contexts.

**Table 2:** Reported conductor properties and measurement context.

Material/Study	Device or Role	Substrate; Dry Film	Sheet Resistance (Ω/sq)	Conductivity (S/m); Provenance	Post-Processing/Interpretation
Orgacon SI-P2000 [26]	HF antenna ink	PET reference: thickness NR; selected paper films 6–7 µm	PET < 0.005; Magno Satin 0.028 ± 0.006	NR for these measured films	150 °C, 10 min; paper coupon result differs from full-antenna resistance
Loctite ECI 1011 [26]	HF antenna ink	PET reference: thickness NR; selected paper films 6–7 µm	PET < 0.005; Magno Satin 0.032 ± 0.002	NR for these measured films	150 °C, 10 min; 0.010–0.034 on selected papers
Metalon JS-A221AE [27]	UHF antenna ink; glass benchmark	Glass reference film 18 µm; antenna on ABS	0.0242, reference film on glass	10^5^–10^7^, process-dependent source-reported range; no per-tag measurement	50 wt% Ag; 35 nm particle size manufacturer-reported; do not assign glass Rs to ABS antenna
Ti_3_C_2_T_x_ MXene [28]	UHF antenna coating	Paper; ≈8.6 µm by SEM	0.3–1.6, local measured values	1.3 × 10^6^ used in simulation; not uniform measured film conductivity	Water dispersion; ≈22 °C drying for 24 h; manual dip coating
Few-layer graphene [16]	UHF transponder antenna	Kapton; thickness NR in screen-printing report	≈5 before/≈3 after annealing, measured	Not reported	265 °C, 10 min; stronger annealing caused delamination
HEC/Mo [29]	Supporting RF shielding; not antenna ink	Capsule coating; thickness NR	0.8 for 1–5 µm Mo at HEC:Mo 1:11	Not reported	Separate supporting example; actual antenna uses zinc foil

**Table 7:** Representative UHF IC-tag validation evidence and conditions for interpreting reported range.

Study and Construction	Result and Evidence Type	Frequency and Link Power	Coupling and Test Setting	Durability and Repeatability	Scope of Validation
Rauter et al. [70] Ag on uncoated paper; AS3213C.4	Transmission up to 2 m; sensing tested at 0.46 m (temperature) and 0.22 m (humidity). Direct demonstrations.	Design: 860–960 MHz; exact frequency of 2 m result NR. EIRP and chip threshold: NR.	Circular reader; orientation NR. Hot plate/climate chamber for sensing; setting of 2 m read NR.	Bending/washing: NR. Humidity-signal SD 1.2%; range repeatability and specimen count NR.	Fabricated tag and controlled sensing tests. The 2 m read is not a validated sensing range or maximum-distance protocol.
Sourkouhi et al. [28] MXene on paper; repurposed IC	Successful read at ≈0.50 m (RSSI ≈ −56 dBm); seven-month recheck at ≈0.35 m. No maximum-range test.	Design context: 800–920 MHz; read-test frequency NR. EIRP and IC sensitivity: NR.	JADAK USB Pro; polarization, orientation and detailed propagation setting NR.	Separate manual-deformation antenna tests; radius/cycles NR. Statistical IC-tag read repeatability NR; washing NR.	Prototype IC communication plus separate antenna tests. ≈97% is impedance-based transfer, not radiation efficiency.
Marindra et al. [31] Inkjet Ag/PET; UCODE G2XL	5.54 → 4.71 m: threshold-extrapolated from fixed-distance measurements; ≈46% less ink, ≈15% less inferred range.	922.5 MHz; reference EIRP 4 W; chip threshold −15 dBm (datasheet).	Linear reader, 8.94 dBi; matched polarization assumed. Anechoic chamber, 1 m separation; mounting NR.	Independent sample count, range repeatability, bending and washing: NR.	Controlled prototype comparison. Source calls ranges measured; the method extrapolates from activation threshold.
Sharif et al. [54] Inkjet PET sticker; Alien H3	Direct indoor maximum 3.2 m. Separate Tagformance result 4.5–5.5 m; extraction protocol NR.	902–928 MHz; 4 W EIRP for indoor test, Tagformance EIRP NR. Chip threshold NR.	Circular reader in indoor test; horizontal/vertical tag orientations and approach directions; mounted on apples.	Almost ten apples; range statistics NR. Curved mounting investigated; repeated bending/washing NR.	Controlled RF and multiple mounted specimens; refrigerator/spoilage application in laboratory, not field qualification.
Jaakkola et al. [16] Graphene; all-graphene or hybrid Al loop	≈6.7 m and ≈11.1 m, respectively: theoretical maximum range from measured threshold.	UHF sweep; peak frequencies NR in extracted prose. EIRP 3.28 W; Monza R6—20 dBm (datasheet).	Anechoic Tagformance cabinet; Styrofoam support. Angular patterns measured; peak-range orientation/polarization and test separation NR.	RF range repeatability, independent specimen count, bending/washing retention: NR.	Fabricated transponders and controlled threshold tests. Hybrid loop changes matching and chip connection; no field-range claim.
Chietera et al. [27] AJP Ag versus conductive filament on ABS	≈7 m AJP versus ≈3 m filament estimates from measured prototype sensitivity.	860–960 MHz sweep; Monza R6; numerical chip threshold and EIRP NR in test description.	Maximum-directivity direction; polarization NR. Laboratory; 3D ABS support, shared commercial Al coupling loop; distance NR.	One shown prototype per route; sample statistics, bending and washing NR.	Comparative prototype test. Not a thin fully printed flexible label or production-yield demonstration.
Wang et al. [68] Flexographic seed + electroless Cu on mineral paper	Successful read/write; absolute maximum range NR in prose. Relative range assessed after bending.	900 MHz design; IC impedance 18—j170 Ω. EIRP and chip sensitivity NR in available main article.	Handheld reader; orientation/polarization and detailed environment NR; paper-supported tag.	800 cycles at 2 and 8 mm radii. Range losses: 25.5% outward/2 mm; 22% inward/2 mm; 4% inward/8 mm. Washing and independent sample count NR.	Fabricated tag and cyclic endurance. Repeated cycles are not independent-tag production repeatability.
Casula et al. [22] AMC-backed tag; supporting architecture	≈5.5 m dielectric, 8 m body and 11 m metal: calculated from simulated gain and matching.	868 MHz design; 30 dBm transmit power + 5.15 dB reader gain; Monza 4–17.4 dBm.	Circular reader model with 1/2 polarization factor; modeled dielectric/body/metal mounting.	Fabrication, bending/washing, repeated measurements: NR.	Simulation only. Supports the isolation mechanism, not measured printed-tag range.
Bansal et al. [23] Assembled 3D patch; supporting architecture	Direct distances: 5.9 m metal; 7.1 m ABS helmet; 7.0 m plastic; 4.9 m foam; 6.1 m wood; 9.3 m glass.	865–867 MHz; 3.28 W EIRP reported in source comparison; Higgs-3–18 dBm.	Circular reader; tag moved away outdoors on stated objects; exact orientation NR.	Independent sample count, range statistics, bending and washing: NR.	Fabricated tag, outdoor distance tests and worker-tracking demonstration. Not a printed-film manufacturing study.

**Table 8:** Environmental evidence distinguished by functional unit, boundary, and type of assessment.

Study and Evidence	Functional Unit and Boundary	Reported Outcome	Interpretation and Limits
Aliakbarian et al. (2024) [78] Attributional LCA; ReCiPe 2016; primary manufacturer inventory	One UHF RFID tag. Raw materials, manufacturing, packaging, transport and end of life; use-phase impacts explicitly excluded.	15.2 g CO_2_-eq for the standard plastic tag versus 11.8 g CO_2_-eq for the paper tag. Study reports approximately 23% reduction; rounded values imply 22.4%.	Architectures differ in mass, material composition and transport. The result does not establish a universal advantage for paper tags. Reader/server use energy is excluded.
Ding et al. (2024) [82] Prospective scenario LCA; ILCD 2.0; modeled technology and demand	Component scenarios: production of one tag or reader, cradle to gate. Supply-chain scenario: 1 kg of battery module produced and delivered, cradle to site, including tags, readers and servers.	No significant paper/PET difference in four main current-tag indicators. Emerging-tag scenarios: 25–70% reductions in most categories; modeled battery supply chains: approximately 3–5% net reductions.	Predictions depend on technology, energy and demand assumptions. These are modeled outcomes, not experimental savings for flexible printed packaging tags.
Zikulnig et al. (2025) [79] Comparative GWP assessment; modeled recycling and biogenic carbon credits	One NFC sensor tag capable of wireless gas monitoring for one day. Raw materials, direct manufacturing energy and end of life; transport, use/readout energy, facilities and certain converting steps excluded.	Reference PLA/Ag/inkjet/incineration: 42 g CO_2_-eq; modeled bio-PE/Cu/screen/recycling: 25.7 g CO_2_-eq per tag. Several variables change together.	Supporting NFC manufacturing evidence. Cracks interrupted copper traces on bio-PE. Lowest modeled footprint does not demonstrate equivalent device reliability or whole-product recyclability.
Pogačar et al. (2024) [20] Experimental deinking and recycled-handsheet assessment	RFID-printed label paper; laboratory INGEDE 11 deinking. No LCA functional unit.	Ink removal was feasible; RFID-containing samples increased residual dirt-particle counts and contaminated area.	Evidence of response to a recycling process. It does not quantify life-cycle impact reduction or establish harmless release of conductive residues.

## Data Availability

No new data were generated or analyzed in this study. Data sharing is not applicable to this article.

## Nomenclature

**Symbol**	**Definition and unit**
λ	Free-space wavelength (m)
$\rho_{pol}$	Forward-link polarization-coupling factor (dimensionless, 0–1)
$P_{EIRP}$	Reader EIRP (W)
$G_{tag}$	Directional tag gain in the reader direction (linear, dimensionless)
$P_{th}$	IC activation threshold (W)
τ	Antenna–chip power-transfer coefficient (dimensionless, 0–1)
$d_{tag}$	Ideal forward-link chip-activation distance (m)
$d_{reader}$	Ideal reader-limited backscatter-detection distance (m)
$d_{system}$	Usable system range (m)
$G_{reader}$	Reader receive gain (linear, dimensionless)
$\sigma_{d}$	Differential radar cross section of the detected modulation component (m^2^)
$S_{r}$	Reader detection threshold for the modulation component (W)
$Z_{ant}$	Antenna input impedance (Ω)
$Z_{chip}$	Chip input impedance (Ω)
$R_{ant}$	Resistive component of antenna impedance (Ω)
$R_{chip}$	Resistive component of chip impedance (Ω)
$X_{ant}$	Reactive component of antenna impedance (Ω)
$X_{chip}$	Reactive component of chip impedance (Ω)
j	Imaginary unit, $\sqrt{-1}$
$R_{rad}$	Radiation resistance (Ω)
$R_{loss}$	Dissipative resistance due to conductor and dielectric losses (Ω)
$L_{eff}$	Effective circuit inductance (H)
$C_{eff}$	Effective circuit capacitance (F)
ω	Angular frequency, 2πf (rad/s)
f	Frequency (Hz)
$R_{trace,DC}$	Direct-current trace resistance (Ω)
$R_{s}$	Sheet resistance (Ω/sq)
l	Trace/current-path length (m)
w	Trace width (m)
g	Gap between adjacent traces (m)
σ	Electrical conductivity (S/m)
h	Conductor thickness (m)
$\eta_{rad}$	Radiation efficiency (dimensionless)
δ	Skin depth (m)
μ	Magnetic permeability (H/m)
Q	Antenna quality factor (dimensionless)
k	Wavenumber (rad/m)
R	Radius of the smallest enclosing sphere (m)
$Z_{j}$	Inverse Ohnesorge number (dimensionless)
ρ	Ink density (kg/m^3^)
γ	Surface tension (N/m)
$d_{n}$	Nozzle characteristic diameter (m)
η	Dynamic viscosity (Pa·s)
$\varepsilon_{r}$	Relative permittivity (dimensionless)
tanδ	Dielectric loss tangent (dimensionless)
$R_{a}$	Arithmetic mean surface roughness (length)
$R_{p}$	Maximum profile peak height/peak roughness parameter (length)
$S_{11}$	Input-port reflection coefficient (dimensionless; commonly reported in dB)
$E_{inc}$	Incident electric field (V/m)
$H_{inc}$	Incident magnetic field (A/m)
$E_{tag}$	Electric field associated with the tag response (V/m)
$H_{tag}$	Magnetic field associated with the tag response (A/m)
$E_{scat}$	Scattered electric field from the tag (V/m)
$H_{scat}$	Scattered magnetic field from the tag (A/m)
$Z_{A}$	Chip/load impedance in modulation state A (Ω)
$Z_{B}$	Chip/load impedance in modulation state B (Ω)
$R_{cond}$	Equivalent conductor-loss resistance (Ω)
$R_{diel}$	Equivalent dielectric-loss resistance (Ω)
**Abbreviation**	**Definition**
3D	Three-dimensional
ABS	Acrylonitrile butadiene styrene
ACA	Anisotropic conductive adhesive
ACNF	Acetylated cellulose nanofiber
AgNP	Silver nanoparticle
AI	Artificial intelligence
AJP	Aerosol jet printing
AMC	Artificial magnetic conductor
bio-PE	Bio-based polyethylene
CO_2_-eq	Carbon dioxide equivalent
DC	Direct current
UV	Ultraviolet
EIRP	Equivalent isotropically radiated power
ERP	Effective radiated power
ETSI	European Telecommunications Standards Institute
GO	Graphene oxide
GWP	Global warming potential
HEC	Hydroxyethyl cellulose
HF	High frequency
IC	Integrated circuit
ILCD	International Reference Life Cycle Data System
INGEDE	International Association of the Deinking Industry
IoT	Internet of Things
LCA	Life-cycle assessment
MXene	Family of two-dimensional transition-metal carbides, nitrides, and carbonitrides
NFC	Near-field communication
NR	Not reported
PEN	Polyethylene naphthalate
PET	Polyethylene terephthalate
PETG	Glycol-modified polyethylene terephthalate
PI	Polyimide
PLA	Polylactic acid
PR	Photoresist
PSO	Particle swarm optimization
PVOH	Polyvinyl alcohol
QR	Quick response
R2R	Roll-to-roll
RCS	Radar cross section
RF	Radio frequency
RFID	Radio-frequency identification
RSSI	Received signal strength indicator
SD	Standard deviation
SEM	Scanning electron microscopy
SIW	Substrate-integrated waveguide
UHF	Ultra-high frequency
USB	Universal Serial Bus
